# JMJD3 facilitates C/EBPβ-centered transcriptional program to exert oncorepressor activity in AML

**DOI:** 10.1038/s41467-018-05548-z

**Published:** 2018-08-22

**Authors:** Shan-He Yu, Kang-Yong Zhu, Juan Chen, Xiang-Zhen Liu, Peng-Fei Xu, Wu Zhang, Li Yan, He-Zhou Guo, Jiang Zhu

**Affiliations:** 1 State Key Laboratory for Medical Genomics, Shanghai Institute of Hematology and Collaborative Innovation Center of Hematology, Rui-Jin Hospital affiliated to Shanghai Jiao-Tong University School of Medicine, Shanghai, 200025 China; 2 School of Life Sciences & Biotechnology, Shanghai Jiao-Tong University, Shanghai, 200040 China

## Abstract

JMJD3, a stress-inducible H3K27 demethylase, plays a critical regulatory role in the initiation and progression of malignant hematopoiesis. However, how this histone modifier affects in a cell type-dependent manner remains unclear. Here, we show that in contrast to its oncogenic effect in preleukemia state and lymphoid malignancies, JMJD3 relieves the differentiation-arrest of certain subtypes (such as M2 and M3) of acute myeloid leukemia (AML) cells. RNA sequencing and ChIP−PCR analyses revealed that JMJD3 exerts anti-AML effect by directly modulating H3K4 and H3K27 methylation levels to activate the expression of a number of key myelopoietic regulatory genes. Mechanistic exploration identified a physical and functional association of JMJD3 with C/EBPβ that presides the regulatory network of JMJD3. Thus, the leukemia regulatory role of JMJD3 varies in a disease phase- and lineage-dependent manner, and acts as a potential oncorepressor in certain subsets of AML largely by coupling to C/EBPβ-centered myelopoietic program.

## Abstract (web summary)

Histone demethylase JMJD3 is known to be oncogenic in preleukemic states and T-cell acute lymphocytic leukemia. Here, the authors show that in some acute myeloid leukemia subsets, JMJD3 can actually act as a potential oncorepressor via mediation of C/EBPβ-centered transcriptional programming.

## Introduction

Classic transcription factors (TFs) associate with histone and DNA modifiers to regulate the transcriptional activation or repression of their specific target genes^1^. Jumonji domain-containing protein D3 (JMJD3) (also named KDM6B) is a family member of the histone H3 lysine 27 tri-methyl (H3K27me3)-specific demethylases that promote gene transcription mainly by acting as the rivals of the polycomb repressive complex 2 (PRC2) that otherwise catalytically add the methyl groups to H3K27^2,3^. In addition, JMJD3 also associates with H3K4 methyltransferase complex to activate gene transcription and other transcriptional co-activators such as SWI/SNF complex to facilitate the transcriptional elongation across the H3K27me3-marked gene body in an enzyme activity-independent manner^4–6^.

Interestingly, unlike another H3K27 demethylase UTX that is constitutively expressed in many types of tissue cells^2,7^, JMJD3 expression is highly inducible by stressful or pathogenic factors including inflammatory cytokines, mitochondrial and oncogenic stress inducers, and by certain normal developmental cues^3,8^. For example, Jmjd3, as induced by lipopolysaccharides (LPS), amyloid and granulocyte-macrophage colony-stimulating factor (GM-CSF), is globally involved in the transcriptional activation of inflammatory genes in M1 macrophages by counteracting the effect of PRC2^9–12^. Jmjd3 is also required for M2 macrophage polarization during the innate immunity response against helminth infection^13^, and involved in TLR2-mediated foamy macrophage formation^14^.

In the aspect of malignant hematopoiesis, an abnormally elevated JMJD3 level in association with an overactivated NF-κb/innate immunity pathway was documented in human CD34^+^ hematopoietic stem/progenitor cells of the myelodysplastic syndrome (MDS)^15^, a preleukemic state that may evolve into acute myeloid leukemia (AML) or acute lymphoid leukemia (ALL). Analogous to this, an oncogenic activity of JMJD3, deeply in association with its role in regulating immune cell differentiation and immunological responses^16,17^, is well documented in lymphoid malignancies^18–20^. Specifically, an oncogenic activity of JMJD3 in the NOTCH1-driven human T-cell acute lymphocytic leukemia (T-ALL) was described^21^. Mechanistically, the NF-κb-induced JMJD3 overexpression in T-ALL cells was found to be essentially associated with NOTCH1 to activate the expression of T cell-specific oncogenic target genes. Nevertheless, what role JMJD3 plays in the maintenance of AML malignancy, probably through collaborating with certain emergency myelopoietic TFs, remains unclear.

## Results

### JMJD3 expressional reduction is correlated with poor prognosis in certain subtypes of AML cases

**Fig. 1:**
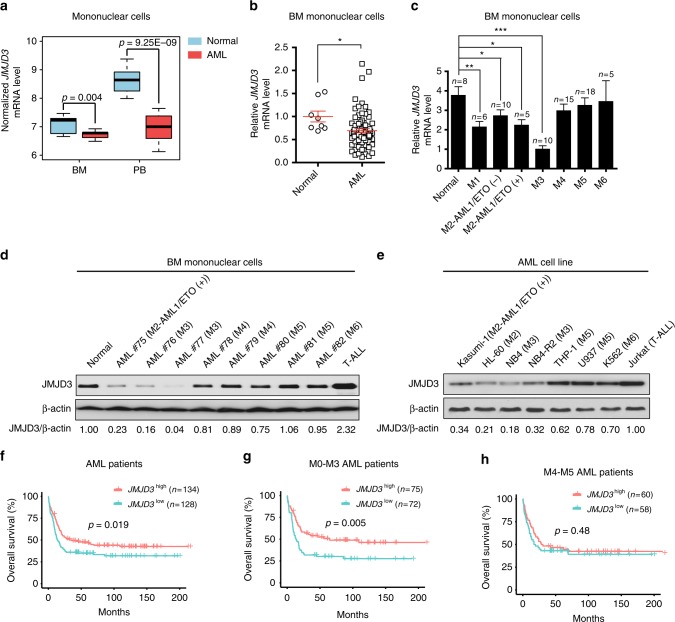
JMJD3 expressional reduction is correlated with poor prognosis in certain subtypes of AML cases. **a** *JMJD3* mRNA levels in BM and PB mononuclear cells of AML patients (including seven BM or 19 PB samples) and in normal counterparts (ten BM and ten PB samples) were compared (raw data retrieved from GEO (GSE9476) dataset). Boxes denote interquartile range, with a line at the median, while whiskers are indicating minimal and maximal observations for each parameter. **b** qRT-PCR assay on the *JMJD3* mRNA level in the AML blasts-enriched primary BM mononuclear cells (*n* = 74) or normal BM mononuclear cells (*n* = 8). **c** qRT-PCR assay on the *JMJD3* mRNA level in the AML blasts-enriched primary BM mononuclear cells including subtype M1 (*n* = 6), M2-AML1/ETO (−) (*n* = 10), M2-AML1/ETO (+) (*n* = 5), M3 (*n* = 10), M4 (*n* = 15), M5 (*n* = 18), M6 (*n* = 5) or normal BM mononuclear cells (*n* = 8). **d** Western blotting assay on JMJD3 protein levels of eight AML blasts-enriched primary BM mononuclear cell samples, a normal BM mononuclear cell sample, and a T-ALL blasts-enriched BM mononuclear cell sample. β-actin level was used as the loading control. **e** Western blotting assay on JMJD3 protein levels of the established leukemia cell lines. **f**–**h** Overall survival of AML patient cohort (**f**), M0, M1, M2, and M3 AML patient cohort (**g**), or M4 and M5 AML patient cohort (**h**) with regard to *JMJD3* mRNA levels. Data are shown as the mean ± SEM; *p < 0.05, **p < 0.01, ***p < 0.001

To understand a possible role of JMJD3 in AML, we firstly explored the NCBI GEO database and also examined the primary bone marrow (BM) samples of 74 AML patients we collected (Supplementary Data 1) to determine whether an abnormal JMJD3 expression existed. In both BM and peripheral blood (PB) mononuclear samples, *JMJD3* mRNA level was significantly reduced in AML blasts compared to normal subjects (Fig. 1a, b). Particularly, the reduction in *JMJD3* mRNA level was most prominent in AML subtypes including M1, M2 (M2 without AML-ETO (AE) fusion protein), M2b (M2 with AE fusion protein), and M3 that show immature features of granulocytic progenitors (Fig. 1c). Consistently, western blotting assay in eight representative AML BM blast samples and seven AML cell lines across M2 to M6 subtypes indicated that among the common AML subtypes, a sharp decrease in JMJD3 protein level most likely occurred in M2 and M3 subtypes, and that among M4 or M5 subtypes, a moderate reduction was not consistently detected (Fig. 1d, e). To test whether different doses of *JMJD3* play a role in AML pathogenesis, we examined a possible association between the *JMJD3* mRNA expression level and the overall survival in a cohort of AML patients from datasets of Verhaak and colleagues^22^. We observed that the survival of patients was positively associated with the *JMJD3* expression level of leukemic blasts, especially in granulocytic subtypes, including M0–M3 but not in monocytic-related subtypes (Fig. 1f, h). Taken together, these results indicate that in contrast to the elevated expression of JMJD3 in MDS or T-ALL cells where it plays an oncogenic role^15,21^, a diminished JMJD3 expression in granulocytic subtypes of AML cells was associated with poor prognosis.

### JMJD3 bears oncorepressor activity in mouse AML models

**Fig. 2:**
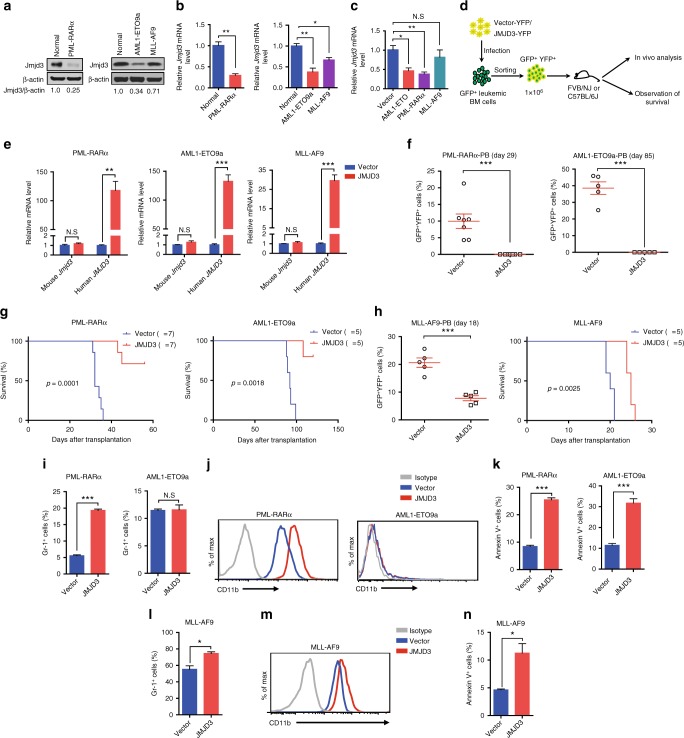
JMJD3 bears oncorepressor activity in mouse AML models. **a**, **b** Western blotting (**a**) or qRT-PCR (**b**) assay on Jmjd3 protein or mRNA level of GFP^+^ murine AML BM myeloid cell samples expressing PML-RARα, AML1-ETO9a, or MLL-AF9, and a normal sorted CD11b^+^ BM myeloid cell sample. **c** AML1-ETO, PML-RARα, or MLL-AF9 was transduced into normal c-Kit^+^ BM cells by retroviral infection, and the mRNA level of *JMJD3* of the transduced GFP^+^ cells was measured by qRT-PCR. **d** Experimental protocol for testing the in vivo proliferative capacity of GFP^+^ BM leukemic cells transduced with YFP^+^ vector control or JMJD3-expressing vector in FVB/NJ or C57BL/6J mice. **e** GFP^+^ murine AML BM cells expressing PML-RARα, AML1-ETO9a, or MLL-AF9 were transduced with YFP^+^ empty vector or JMJD3-expressing vector, and the mRNA levels of mouse *Jmjd3* or human *JMJD3* were measured by qRT-PCR. **f** The percentages of PML-RARα (left panel) or AML1-ETO9a (right panel) GFP^+^YFP^+^ cells in the peripheral blood of syngeneic recipients after they had been transduced with YFP^+^ empty vector or JMJD3-expressing vector. **g** Kaplan–Meier curves are shown for syngeneic mice transplanted with GFP^+^ AML (left panel, PML-RARα^+^ cells; right panel, AML1-ETO9a^+^ cells) BM cell transduced with YFP^+^ empty vector or JMJD3-expressing vector. **h** The percentages of MLL-AF9^+^ GFP^+^YFP^+^ cells in the peripheral blood (left panel) and the survival (right panel) of the syngeneic recipients transduced with YFP^+^ empty vector or JMJD3-expressing vector. **i**, **j** Flow cytometric analyses of Gr-1 expression (**i**) or CD11b expression (**j**) on PML-RARα^+^ (left panel) or AML1-ETO9a^+^ (right panel) leukemia BM cells transduced with YFP^+^ empty vector or JMJD3-expressing vector and cultivated for 72 h. **k** Flow cytometric analyses of Annexin V staining on PML-RARα^+^ (left panel) or AML1-ETO9a^+^ (right panel) leukemia BM cells transduced with YFP^+^ empty vector or JMJD3-expressing vector. **l**–**n** Flow cytometric analyses of Gr-1 expression (**l**), CD11b expression (**m**), or Annexin V staining (**n**) on MLL-AF9^+^ BM cells transduced with YFP^+^ empty vector or JMJD3-expressing vector in vivo. Data are shown as the mean ± SEM; *p < 0.05, **p < 0.01, ***p < 0.001

Next, we employed three mouse AML models including one granulocytic M2b subtype expressing a human AML1-ETO9a (AE9a) fusion protein and one granulocytic M3/APL subtype expressing PML-RARα (PR) fusion protein as well as one monocytic M5 subtype expressing MLL-AF9 (MF9) fusion protein to test whether JMJD3 possessed a genuine oncorepressor potential in AML^23–25^. Consistent with the observations made in human AML cells (Fig. 1c, e), Jmjd3 mRNA and protein levels in the BM blasts of M2b model and M3 model were greatly decreased compared to normal counterparts, while Jmjd3 mRNA and protein levels in M5 blasts were only moderately reduced (Fig. 2a, b). The genomic browser tracks of the chromatin immunoprecipitation sequence (ChIP-seq) analyses in human M2b Kasumi cells, M3/APL NB4, and UPR9 cells, and M5 MLL-AF9 cells^26–28^ indicated that AE or PR fusion protein occupied the gene locus of *JMJD3* while MF9 did not (Supplementary Fig. 1a), implicating *JMJD3* as a direct target of the oncogenic proteins for two granulocytic subtypes. Accordingly, the introduction of the AE or PR but not MF9 into the normal mouse c-Kit^+^ BM progenitors immediately decreased the *Jmjd3* mRNA level (Fig. 2c).

Then we lentivirally transduced the primary leukemic blastic BM samples with empty vector or JMJD3 expression cassette in a short-term culture, and transplanted the purified transduced GFP^+^YFP^+^ AML cells into the syngeneic recipients (Fig. 2d). JMJD3 overexpression potently inhibited the in vivo repopulation of two granulocytic subtypes of AML cells (Fig. 2e, f and Supplementary Fig. 1b), and greatly elongated the recipients’ survival (Fig. 2g). Virtually, deaths of a few recipients were from the outgrowth of GFP^+^YFP^−^ leukemia cells that did not express JMJD3 (Supplementary Fig. 1c). On the other hand, JMJD3 overexpression only moderately inhibited the repopulation kinetics of M5 cells in vivo (Fig. 2h and Supplementary Fig. 1d). The cellular mechanistic analyses of the in vitro cultivated M2b and M3 cells and in vivo retrieved M5 cells indicated that JMJD3 exerted strong differentiation- and apoptosis-induction effects in M3 model, a strong apoptosis-induction effect in M2b model, and moderate differentiation- and apoptosis-induction effects in M5 model (Fig. 2i, n and Supplementary Fig. 1e, j). Therefore, JMJD3 exerts a potential anti-AML activity in a subtype and oncogenic fusion protein-dependent manner.

### JMJD3 exhibited anti-human AML activity in a demethylase-dependent manner

**Fig. 3:**
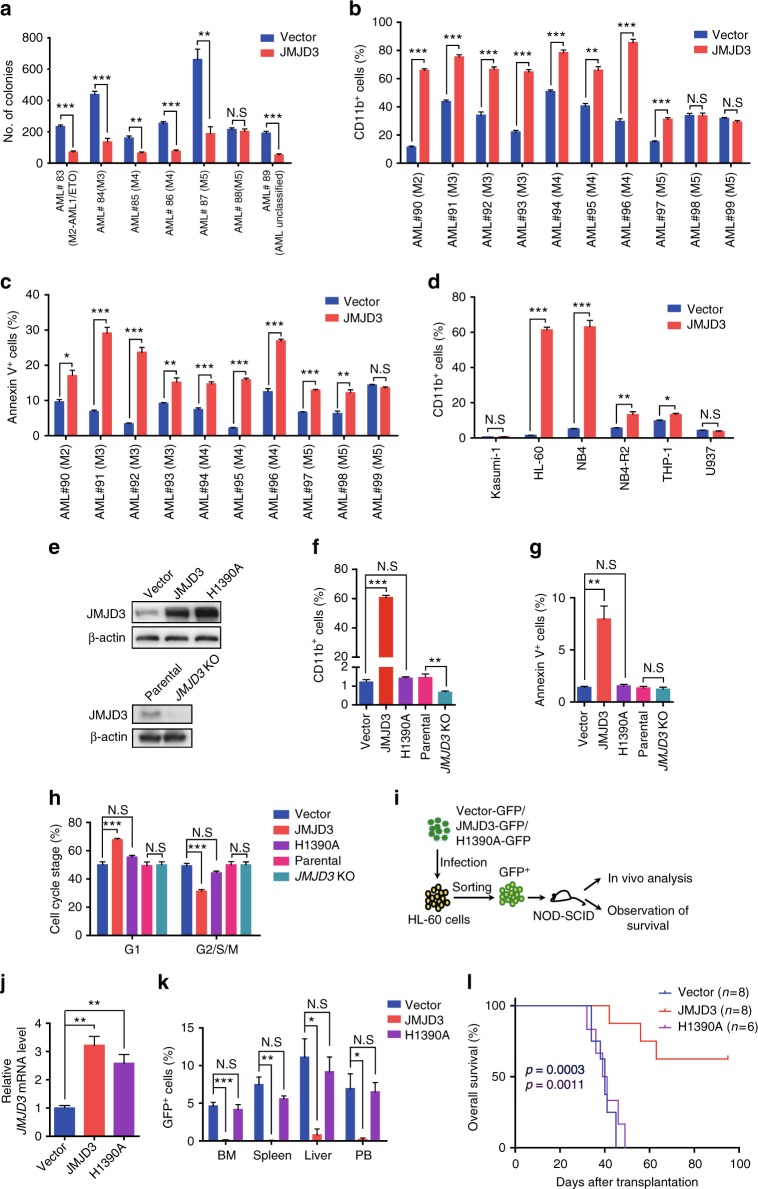
JMJD3 exhibited anti-human AML activity in a demethylase-dependent manner. **a** The number of the leukemic colony forming units for seven primary AML blast samples transduced with control vector or JMJD3-expressing vector. **b**, **c** Flow cytometric analyses of CD11b expression (**b**) or Annexin V staining (**c**) on ten primary AML blast samples transduced with control vector or JMJD3-expressing vector. **d** Flow cytometric analyses of CD11b expression on six AML cell lines transduced with control vector or JMJD3-expressing vector. **e** Western blotting assay on JMJD3 protein in HL-60 cells transduced with vector control, JMJD3-, or H1390A mutant-expressing vector (upper panel), and in parental and *JMJD3* KO HL-60 cell lines (bottom panel). **f**–**h** Flow cytometric analyses of CD11b expression (**f**), Annexin V staining (**g**), and cell cycles (**h**) in HL-60 cells transduced with vector control, JMJD3-, or H1390A mutant-expressing vector, or in parental or *JMJD3* knockout HL-60 cells. **i** Experimental protocol for testing the in vivo proliferative capacity of HL-60 cells transduced with vector control, JMJD3-, or H1390A mutant-expressing vector in NOD/SCID mice; 5 × 10^6^ transduced GFP^+^ HL-60 cells were intravenously transplanted into each NOD/SCID mouse. **j** qRT-PCR assay on the *JMJD3* mRNA level of GFP^+^ HL-60 cells isolated from the BM at 35 days after transplantation. **k** The percentages of GFP^+^ cells in BM, spleen, liver, and PB at 35 days after transplantation. **l** Kaplan–Meier survival curves of NOD/SCID mice injected with HL-60 cells transduced with vector control, JMJD3-, or H1390A mutant-expressing vector; *p* = 0.0003 (blue), vector versus JMJD3; *p* = 0.0011 (purple), H1390A versus JMJD3. Data are shown as the mean ± SEM; *p < 0.05, **p < 0.01, ***p < 0.001

In accordance with its role in mouse AML models, the lentiviral vector-delivered JMJD3 expression-cassette resulted in JMJD3 overexpression and decreased the colony-forming potential of the primary leukemic blasts in six out of seven cases (in one M2b, one M3, two M4, and one unclassified AML case but only in one out of two M5 cases) (Fig. 3a and Supplementary Fig. 2a). Likewise, JMJD3 overexpression showed myeloid differentiation-induction and apoptosis-induction effect in one M2 (without AE), three M3/APL, two M4, and one or two out of three M5 human primary blastic BM samples (Fig. 3b, c and Supplementary Fig. 2b, d). Nevertheless, JMJD3 knockdown in five primary AML blastic samples (one M2 without AE, one M3, one M4, and two M5) decreased CD11b level in four cases except one M5 but not significantly influenced their survival status (Supplementary Fig. 2e, h), indicating a genuine regulatory role of JMJD3 on the differentiation status of AML blasts.

Strikingly, a JMJD3 overexpression-induced CD11b expression was prominent in a M2/M3 cell line HL-60 (without AE and PR) and NB4 cell line (with PR) as compared to that detected in M2b Kasumi cells, all-trans retinoid acid (ATRA)-resistant M3 NB4-R2 cells, and M5 THP-1 (with MF9) and U937 cells, whereas apoptosis-induction effect was apparent in all tested cell lines except U937 cells (Fig. 3d and Supplementary Fig. 3a, b). Thereafter, we mainly used HL-60 cells or/and NB4 cells to further characterize the relevant mechanisms of JMJD3 in regulating AMLs. As evidenced by western blotting assay and flow cytometric analyses of myeloid differentiation antigen CD11b expression, cell survival, and cell cycle (Fig. 3e, h and Supplementary Fig. 3c, e), the forced expression of WT JMJD3 but not H1390 mutant or empty vector potently drove the myeloid differentiation of AML cells in a demethylase-dependent manner (by 50-fold), to be accompanied by the moderately reduced survival (by seven-fold) and proliferation of leukemia cell. Conversely, the *JMJD3* knockout at least moderately hindered the CD11b expression of HL-60 (Fig. 3e, h and Supplementary Fig. 3c, e). Critically, the overexpression of JMJD3, but not the empty vector or a catalytically inactive JMJD3 mutant (H1390A), prolonged the survival of the NOD/SCID mouse recipients intravenously inoculated with the transduced GFP^+^ HL-60 cells (Fig. 3i, l). As expected, the much more GFP^+^ leukemia cells were readily detected in BM, spleen, liver, and PB of the mice transplanted with leukemia cells harboring the empty vector or overexpressing H1390A but not with leukemia cells overexpressing JMJD3 (Fig. 3k and Supplementary Fig. 3f). Overall, these observations demonstrated a potential oncorepressor activity of JMJD3 enzyme in vivo and in vitro in the setting of certain subtypes of AML such as M2 and M3, which was most likely in association with the forced myeloid differentiation as well as the reduced survival and proliferation of AML blasts.

### JMJD3 induces the expression of key myelopoietic regulators in human AML cells

**Fig. 4:**
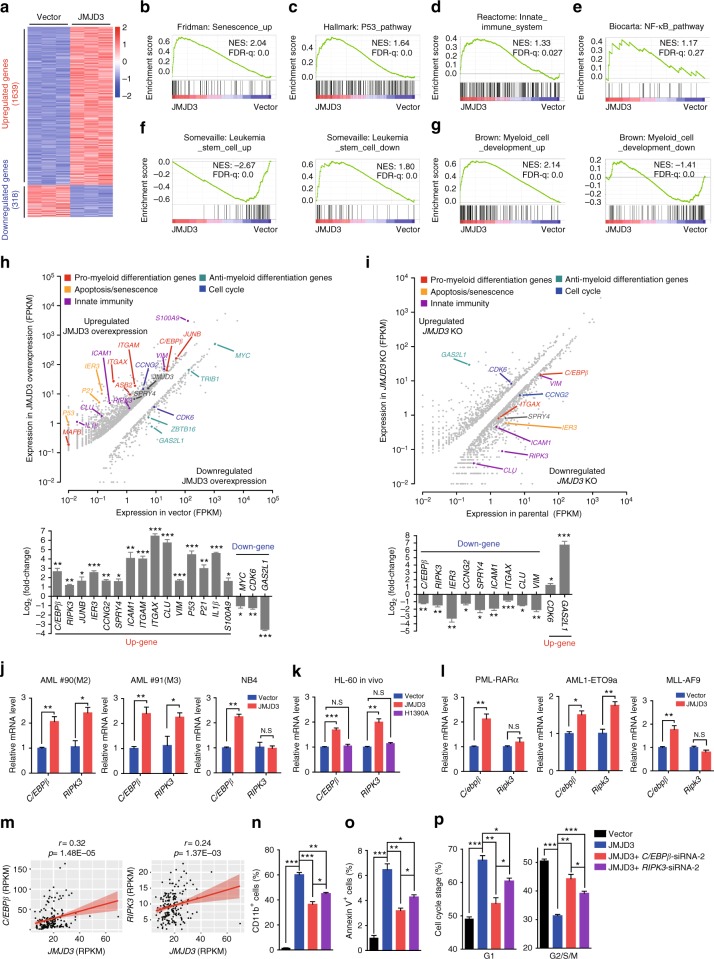
JMJD3 induces the expression of key myelopoietic regulators in AML cells. **a** Heatmap showing the differentially expressed genes between HL-60 cells transduced with control or JMJD3-expressing vector (fold change ≥2, *p* < 0.05). **b**–**g** GSEA of the expressing profile of HL-60 cells transduced with control or JMJD3-expressing vector using a senescence-associated upregulated signature (**b**), a p53 pathway-associated signature (**c**), an innate immune system-associated signature (**d**), a NF-κB pathway-associated signature (**e**), a leukemic stem cell (LSC)-associated upregulated or downregulated signature (**f**), and a myeloid cell development-associated upregulated or downregulated signature (**g**). **h** Differential expression analyses of HL-60 cells upon overexpression of JMJD3 (fold change ≥2, *p* < 0.05) (top panel), and validation of the mRNA levels of a number of genes by qRT-PCR (bottom panel). FPKM, fragments per kilobase of transcript per million fragments mapped. **i** Differential expression analysis upon knockout of *JMJD3* in HL-60 (fold change ≥1.5, *p* < 0.05) (top panel), and validation of the mRNA levels of a number of genes by qRT-PCR (bottom panel). **j** Two primary AML blasts and the NB4 cells were transduced with empty vector or JMJD3-expressing vector, and the mRNA levels of *C/EBPβ* or *RIPK3* were measured by qRT-PCR. **k** NOD/SCID mice injected with HL-60 cells transduced with vector control, JMJD3-, or H1390A mutant-expressing vector, and qRT-PCR assay on the *C/EBPβ* or *RIPK3* mRNA level of GFP^+^ HL-60 cells isolated from the BM at 35 days after transplantation. **l** GFP^+^ murine AML BM cell samples from PML-RARα-, AML1-ETO-, or MLL-AF9-expressing transgenic mice were transduced with empty vector or JMJD3-expressing vector, and the mRNA levels of *C/EBPβ* or *RIPK3* were measured by qRT-PCR. **m** Correlated mRNA levels of *C/EBPβ* or *RIPK3* with those of *JMJD3* in 179 AML patients (raw data from TCGA database). **n**–**p** HL-60 cells transduced with vector control or JMJD3-expressing vector were further treated with NC siRNA, *C/EBPβ* siRNA, or *RIPK3* siRNA. Flow cytometric analyses of CD11b expression (**n**), annexin V staining (**o**), and cell cycle status (**p**). Data are shown as the mean ± SEM; *p < 0.05, **p < 0.01, ***p < 0.001

The demethylase activity-dependent suppressing role of JMJD3 in HL-60 cells indicated that this effect was largely executed through the modulation of the transcriptional program of AML cells. Therefore, we explored the JMJD3 overexpression- and *JMJD3* knockout-caused mRNA expressional alterations to identify the responsible target genes (Supplementary Data 2–3). In agreement with the documented role of JMJD3 as a transcriptional co-activator, RNA sequencing (RNA-seq) showed that the number of the upregulated genes versus that of the downregulated ones by JMJD3 overexpression in HL-60 cells was 1639:318 (Fig. 4a). In line with the previous observation^29,30^, the gene set enrichment analysis (GSEA) revealed significant enrichment of senescence genes and P53 pathway genes in JMJD3-overexpressed HL-60 cells (Fig. 4b, c). Nevertheless, the transcription of *INK4a-ARF* locus was not altered by JMJD3 overexpression (Supplementary Fig. 4a)^29^. Albeit the signatures of inflammatory response and innate immunity pathways were enriched in JMJD3-overexpressed HL-60 cells (Fig. 4d and Supplementary Fig. 4b), JMJD3 overexpression did not elevate the mRNA level of *P65* subunit of NF-κB pathway or those of NOTCH1 targets *HEY1* and *HES5* (Fig. 4e and Supplementary Fig. 4c, e)^15,21^. Interestingly, JMJD3 overexpression also negatively regulated the expression of a leukemia stem cell (LSC) gene signature and that of a hematopoietic early progenitor gene signature (Fig. 4f and Supplementary Fig. 4f left panel). Conversely, JMJD3 overexpression positively modulated the expression of the gene signatures of the myeloid development and mature hematopoietic cells (Fig. 4g and Supplementary Fig. 4f right panel). GSEA also showed a positive enrichment for the gene signature associated with apoptosis, and a negative enrichment for the gene signature associated with cell cycle in JMJD3-overexpressed HL-60 cells (Supplementary Fig. 4g, h). Likewise, gene ontology (GO) analyses revealed that the genes upregulated upon JMJD3 overexpression were enriched in functional categories linked to hematopoietic differentiation and apoptosis (Supplementary Fig. 4i). Similarly, JMJD3 overexpression in NB4 cells downregulated the expression of the LSC gene signature, the hematopoietic early progenitor signature and the cell cycle signature, but upregulated the expression of the mature hematopoietic cell signature without activating the pathways of senescence, innate immunity, NF-κB, or the NOTCH1 targets *HEY1* and *HES5* (Supplementary Fig. 4j, r). In line with the observations mentioned above (Fig. 3), these analyses confirmed that the enhanced myeloid differentiation, in companion of the suppressed LSC program but not the enhanced apoptosis and cell cycle arrest, represented the major effector pathways of JMJD3 overexpression in AML cells.

As expected, JMJD3 overexpression or *JMJD3* knockout in HL-60 cells altered the mRNA levels of a number of key regulators of myeloid differentiation, apoptosis/senescence, cell cycle and innate immunity in a reverse way, which was confirmed by quantitative polymerase chain reaction (qPCR) assays (Fig. 4h, i). The overlapping of these two pools identified two highly sensitive responsive genes: *C/EBPβ*—a key TF-mediating emergency myelopoiesis, and *RIPK3*—a key suppressor of AML malignancy through activating IL-1β-related innate immunity pathway^31–34^. In agreement, JMJD3 overexpression upregulated the *C/EBPβ* mRNA level in human AML cells cultivated in vitro or HL-60 cells repopulated in the NOD/SCID mice as well as in three subtypes of mouse AML cells, and also the *RIPK3* mRNA level in these cells except NB4 cells, the mouse M3/APL cells and the mouse M5 cells expressing MF9 (Fig. 4j, l and Supplementary Fig. 4s). The in silico analysis showed that *JMJD3* mRNA level was positively correlated with *C/EBPβ* or *RIPK3* mRNA level among primary AML blast samples (Fig. 4m), indicating that the regulation of *C/EBPβ* or *RIPK3* by JMJD3 was generally present in AML cells. Moreover, *C/EBPβ* or *RIPK3* knockdown exerted a reversing effect on the JMJD3 overexpression-induced myeloid differentiation, cell death, and cell cycle arrest, with C/EBPβ showing a stronger effect than RIPK3 (Fig. 4n, p and Supplementary Fig. 4t, x). Taken together, these results indicated that *C/EBPβ* and *RIPK3* represent two major targets of JMJD3 to mediate the myeloid differentiation, cell cycle arrest, and cell death of AML blasts.

### JMJD3 regulates dynamic H3K4me3 and H3K27me3 modifications in the promoter of myelopoietic genes

**Fig. 5:**
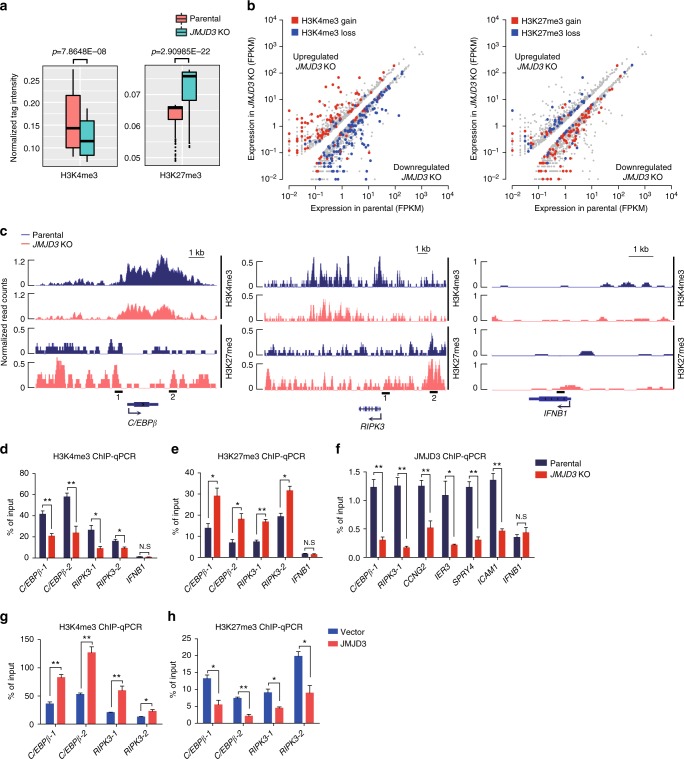
JMJD3 regulates dynamic H3K4me3 and H3K27me3 modifications in the promoter of myelopoietic genes. **a** Box plots showing the differences of H3K4me3 (left) and H3K27me3 (right) occupancies around the promoter (with 3 kb upstream or downstream of the TSS) of parental and *JMJD3* knockout HL-60 cells. **b** The loci of the downregulated genes in JMJD3 deficiency exhibit a decrease in H3K4me3 (blue dots, left panel) but an increase in H3K27me3 level (red dots, right panel). FPKM, fragments per kilobase of transcript per million fragments mapped. **c** Genome browser tracks representing the binding sites of H3K4me3 and H3K27me3 at the *C/EBPβ* or *RIPK3* gene locus in parental and *JMJD3* knockout HL-60 cells. **d**, **e** ChIP–qPCR assay for H3K4me3 (**d**) and H3K27me3 (**e**) at the *C/EBPβ*, *RIPK3* or *IFNB* gene locus in parental and *JMJD3* knockout HL-60 cells. **f** ChIP–qPCR assay for JMJD3 occupancy at a number of gene loci in parental and *JMJD3* knockout HL-60 cells. **g**, **h** ChIP–qPCR assay for H3K4me3 (**g**) and H3K27me3 (**h**) at the *C/EBPβ* or *RIPK3* gene locus in HL-60 cells transduced with empty vector or JMJD3-expressing vector. Data are shown as the mean ± SEM; *p < 0.05, **p *<* 0.01

Then we performed ChIP-seq and ChIP–qPCR assays to compare the possible alterations in H3K4me3 and H3K27me3 of the JMJD3-regulated genes after JMJD3 knockout in HL-60 cells. The JMJD3 loss globally reduced the H3K4me3 abundance but increased the H3K27me3 abundance around numerous promoters in HL-60 cells (Fig. 5a). As expected, most of the downregulated genes in the *JMJD3*^−/−^ HL-60 cells were in association with a reduced H3K4me3 but an increased H3K27me3 around the promoter within 3 kb upstream or downstream of the transcription start site (TSS) (Fig. 5b). As shown by the genomic browser tracks and validated by ChIP–qPCR, the reduced H3K4me3 and increased H3K27me3 were evident around the promoters of the key myelopoietic regulators such as *C/EBPβ*, *IER3*, *CCNG2*, and *SPRY4* as well as the innate immunity component such as *RIPK3* and *ICAM1*, but not in the gene locus of *IFNB1* that serves as a negative control of JMJD3 target genes (Fig. 5c, e and Supplementary Fig. 5a, c. See also Fig. 4i). In parallel, ChIP–qPCR assay on the potential JMJD3-binding sites, where the H3K27me3 abundance was significantly altered in *JMJD3*^−/−^ HL-60 cells, showed that JMJD3 knockout removed the occupancy of JMJD3 (Fig. 5f). These results indicated that a number of myelopoietic regulators and innate immunity components in AML cells belonged to the direct target genes of JMJD3. Expectedly, JMJD3 overexpression increased H3K4me3 but decreased H3K27me3 around these potential JMJD3-occupying sites (Fig. 5g, h and Supplementary Fig. 5d, e).

### JMJD3 upregulates and partners with C/EBPβ to regulate the expression of key myelopoietic regulators in AML cells

**Fig. 6:**
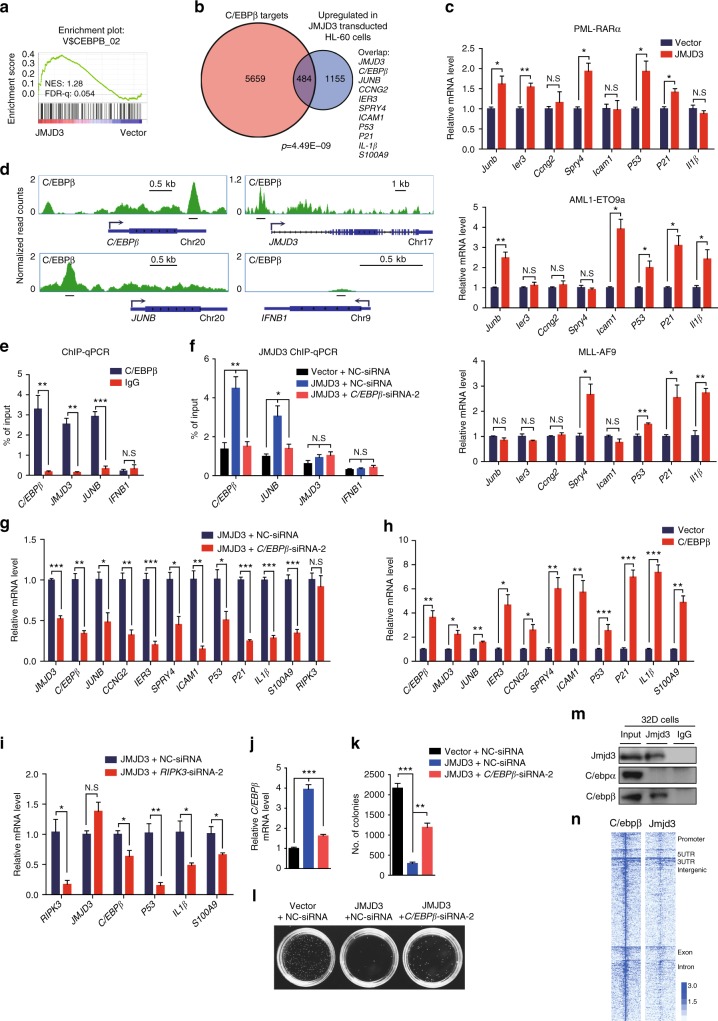
JMJD3 upregulates and partners with C/EBPβ to regulate the expression of key myelopoietic genes in AML cells. **a** GSEA of the expressing profile of HL-60 cells transduced with control or JMJD3-expressing vector using a *C/EBPβ* target genes-associated signature. **b** Venn diagram showing the overlap between *C/EBPβ* promoter targets in C/EBPβ-overexpressed HL-60 cells (red, see also Supplementary Data 4) and the upregulated genes in JMJD3-overexpressed HL-60 cells (gray, see also Supplementary Data 2) with a *p* value calculated by hypergeometric test. **c** GFP^+^ murine AML BM cell samples from PML-RARα- (upper panel), AML1-ETO9a- (middle panel), or MLL-AF9- (low panel) expressing transgenic mice were transduced with an empty vector or JMJD3-expressing vector, and the mRNA levels of a number of genes were measured by qRT-PCR. **d** Genome browser tracks representing the binding sites of C/EBPβ at the *C/EBPβ*, *JUNB*, *JMJD3* or *IFNB1* gene locus. **e** ChIP–qPCR assay for C/EBPβ or IgG occupancy at the *C/EBPβ*, *JUNB*, *JMJD3* or *IFNB1* gene locus in C/EBPβ-overexpressed HL-60 cells. **f** ChIP–qPCR assay for JMJD3 occupancy at the *C/EBPβ*, *JUNB, JMJD3* or *IFNB1* gene locus for NC or *C/EBPβ* knockdown HL-60 cells transduced with control or JMJD3-overexpressed vector. **g** qRT-PCR assay on the mRNA levels of a number of genes when HL-60 cells were transduced with control or C/EBPβ-expressing vector. **h** qRT-PCR assay on the mRNA levels of a number of genes when JMJD3-overexpressed HL-60 cells were transduced with or without *C/EBPβ* siRNA. **i** qRT-PCR assay on the mRNA levels of a number of genes when JMJD3-overexpressed HL-60 cells were transduced with or without *RIPK3* siRNA. **j**–**l** The number and typical pictures of the colonies for NC or *C/EBPβ* knockdown HL-60 cells that had been transduced with control or JMJD3-overexpressing vector. **m** 32D cell lysates were immunoprecipitated with Jmjd3 antibody and subsequently subjected to western blot with Jmjd3, C/ebpα or C/ebpβ antibody: IgG served as negative IP control. **n** Heatmap representation of C/ebpβ and Jmjd3 tag density centered on C/ebpβ ChIP-seq peaks in 32D cells. Data are shown as the mean ± SEM; *p < 0.05, **p < 0.01, ***p < 0.001

Then we sought for the principal TFs that recruited JMJD3 to the regulatory regions of these key myelopoietic regulators, P53–P21 axis, and innate immunity components. Interestingly, GSEA exhibited an enrichment of C/EBPβ-bound promoter targets in JMJD3-overexpressed HL-60 cells (Fig. 6a). Besides, we did an analysis of the promoter targets of C/EBPβ in HL-60 cells by ChIP-seq (Supplementary Data 4), and noticed that about 1/3 of JMJD3-upregulated genes were also the direct target genes of C/EBPβ, displaying a significant correlation (*p* = 4.49 × 10^−9^, Fig. 6b). Interestingly, the enhanced expression of these C/ebpβ target genes was also recapitulated in the mouse AML cells when JMJD3 was overexpressed (Fig. 6c and also see Fig. 4l). Specifically, these overlapped genes included *C/EBPβ* and *JMJD3* themselves, other myelopoietic regulators and innate immunity components such as *JUNB*, *IER3*, *CCNG2*, *SPRY4*, *ICAM1*, *P53-P21* axis, *IL-1β*, and *S100A9* (but not *RIPK3*) (Fig. 6b, d, e and Supplementary Fig. 6a, b), thus indicating C/EBPβ as one of the principle TFs that recruited JMJD3 to the target promoters and the existence of a positive feedback mechanism of JMJD3 regulatory effect through activating *C/EBPβ* expression in AML cells. In support of this, C/EBPβ knockdown almost abolished the occupancy of JMJD3 on the promoter of these target genes (Fig. 6f and Supplementary Fig.6c), and significantly dampened the mRNA-elevating effect of JMJD3 on these genes (Fig. 6g). Consistently, *C/EBPβ* overexpression in HL-60 cells elevated the mRNA levels of the same set of target genes (except *RIPK3*) as regulated by JMJD3 (Fig. 6h). Moreover, knockdown of *JUNB*, a potent oncorepressor of myeloid leukemia^35,36^, at least partially mimicked the role of *C/EBPβ* knockdown in reversing the differentiation-promoting effect of JMJD3 overexpression (Supplementary Fig. 6d, e). On the other hand, *RIPK3* knockdown dampened the JMJD3 overexpression-induced upregulation of *C/EBPβ*, *IL-1β*, and *S1009A* but not *JMJD3* itself, indicating an upstream role of RIPK3 in facilitating C/EBPβ induction or activation (Fig. 6i). Collectively, these observations suggested a nodal role of the C/EBPβ and JMJD3 partnership in mediating the AML-suppressing effect of JMJD3. Verifying this, *C/EBPβ* knockdown significantly restored the colony forming capacity of HL-60 cells as repressed by JMJD3 overexpression (Fig. 6j, l).

Similar to what we observed in the expressional regulation of JMJD3 in AML cells, *C/EBPβ* mRNA level was decreased in primary AML blasts compared to normal counterparts (Supplementary Fig.6f), and ChIP-seq analysis of AML cells and transfection experiment of normal c-Kit^+^ BM progenitors indicated that the transcription of *C/EBPβ* was likely repressed by oncogenic proteins such as PML-RARα (Supplementary Fig. 6g, h). Nevertheless, in murine non-malignant myeloid progenitor cell line 32D cells, the expressions of C/ebpβ and Jmjd3 were readily detectable by western blotting. As expected, co-immunoprecipitation (co-IP) assay showed that endogenous Jmjd3 physically associated with C/ebpβ but not C/ebpα, another major myeloid TF (Fig. 6m), which was confirmed by co-IP assay of 293T cells expressing human C/EBPβ and JMJD3 (Supplementary Fig. 6i). Virtually, ChIP-seq profiling of C/ebpβ and Jmjd3 binding sites across the genome of 32D cells revealed that C/ebpβ and Jmjd3 were highly correlated on intergenic and intragenic regions, suggesting that C/ebpβ and Jmjd3 coregulate the expression of a great number of genes potentially transcribed in the non-malignant myeloid progenitors (Fig. 6n). Taken together, these results indicated that the regulatory activity of JMJD3 during myelopoiesis was at least partially accomplished by forming a special partnership with C/EBPβ in which the autocrine induction of these two genes by either of them may rapidly bring about a strong anti-AML effect.

### C/EBPβ/JMJD3 induction participates in ATRA-induced myeloid differentiation of AML cells

**Fig. 7:**
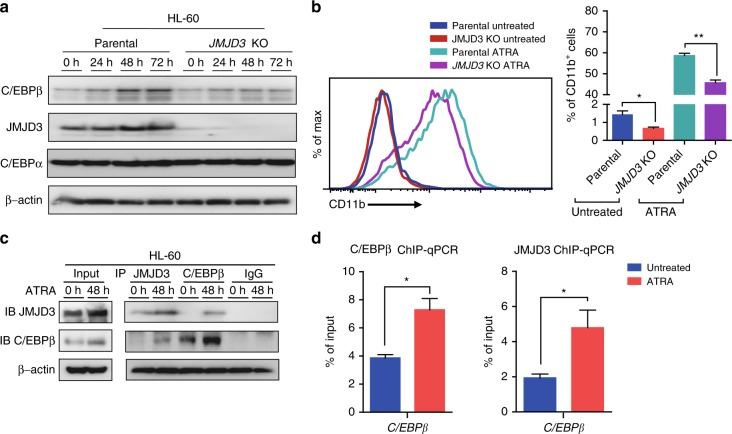
C/EBPβ/JMJD3 induction participates in ATRA-induced myeloid differentiation of AML cells. **a** HL-60 cells were treated with 1 µM ATRA for the indicated times. The protein levels of C/EBPβ, JMJD3, and C/EBP*α* were measured by western blotting, and β-actin was used as a loading control. **b** Flow cytometric analyses of CD11b expression of HL-60 parental cells or JMJD3 knockout HL-60 cells treated with or without 1 µM ATRA for 48 h. **c** Hl-60 cells were treated with 1 µM ATRA for 48 h. Cell lysates were then co-immunoprecipitated with JMJD3 antibody, C/EBPβ antibody or control IgG, to be followed by immunoblot with anti-C/EBPβ or anti-JMJD3 antibody as indicated. **d** ChIP–qPCR for JMJD3 or C/EBPβ occupancy at the *C/EBPβ* gene locus for HL-60 cells treated with or without 1 µM ATRA for 24 h. Data are shown as the mean ± SEM; *p < 0.05, **p < 0.01

ATRA-induced myeloid differentiation of multiple subtypes of AML cells (especially M2 and M3/APL subtypes) inhibits their leukemogenic potential in patients and in patient-derived xenografting models^37,38^. C/EBPβ induction has been shown to participate in the ATRA-induced myeloid differentiation of AML cells^39^, while what epigenetic factor(s) cooperated with C/EBPβ in effecting myeloid differentiation remained unclear. In line with the above observations that C/EBPβ partnered with JMJD3, upon the forced JMJD3 overexpression, to regulate the myeloid differentiation program of AML cells, western blotting assay showed that ATRA induced a significant induction of JMJD3 in parallel to the induction of C/EBPβ in HL-60 cells, and that JMJD3 deficiency compromised the ATRA-induced C/EBPβ upregulation and myeloid differentiation (Fig. 7a, b). As expected, co-IP and ChIP–qPCR assays on *C/EBPβ* locus demonstrated that ATRA signaling enhanced the C/EBPβ/JMJD3 association and their occupancy on *C/EBPβ* promoter (Fig. 7c, d).

## Discussion

In the case of T-ALL and MDS, an oncogenic role of JMJD3 has been well documented, which was largely attributed to its specific partnership with NF-κB and NOTCH^15,21^, two TFs whose overactivations are highly associated with the induction of multiple types of inflammatory cytokines and the proliferation of T cells^3,40^. On the other hand, although JMJD3 is well-documented to mediate the oncogenic stress-induced cell senescence via activating *INK4* gene locus (*INK4b-ARF-INK4a*) and/or facilitating the regulatory activity of P53 on its target genes in non-malignant fibroblasts^29,30,41–43^, a corresponding oncorepressor activity of JMJD3 in human malignancies via cell senescence induction has not been established. Virtually, it was recently shown that JMJD3-induced *INK4a*/*P16* expression was beneficial to the survival of cervical carcinoma cells^44^. Nevertheless, JMJD3 deficiency was reported to promote malignant progression of human pancreatic carcinoma by decreasing the expression of C/EBPα, a potential inhibitory partner of E2F1^45,46^. Moreover, an elevated H3K27me3 level in association with JMJD3 insufficiency promoted the dedifferentiation of hypoxic ^V600E^*BRAF* melanoma cells and rendered them resistant to BRAF inhibitor^47^. These observations implicate the existence of uncharacterized regulatory effects of JMJD3 on cell differentiation, survival, and proliferation, whose deficiency contributes to the carcinogenesis in a tumor cell type-dependent manner.

How the origin and progression of malignant myelopoiesis are associated with the C/EBPβ-centered myelopoietic program is an unsolved issue^48–50^. In this study, we provide evidence that repression of C/EBPβ/JMJD3 partnership probably represents an essential oncogenic event for the maintenance of leukemic malignancy in certain subtypes of AML (mostly M2 and M3), and their overexpression drives these AML cells to undergo myeloid differentiation and lose their capacity to re-establish leukemia in vivo. In this regard, in parallel to the observation that JMJD3 expression is highly inducible by ATRA^51^, C/EBPβ has been shown to mediate ATRA-induced differentiation of AML cells^39,52^, and to suppress the malignancy of chronic myeloid leukemia blasts by inducing their myeloid maturation^53,54^. Moreover, in parallel to the fact that JMJD3 in myeloid cells is highly inducible during innate-immunity response^3^, C/EBPβ has been shown to specifically participate in the innate immunity-related emergency myelopoiesis but not the steady myelopoiesis^31,55^. Likewise, a few major components of innate immunity response such as TLR8 and IRF8 have been shown to modulate the malignant origin and maintenance of AML cells^56,57^. Furthermore, the progenitors of emergency myelopoiesis were recently shown to share the same clustering proliferative feature with the malignant myeloid blasts^58^. Taken together, these observations implicate a possible regulatory effect of emergency myelopoietic program on the maintenance of AML malignancy.

In many types of inflammatory progression, the expression of JMJD3 is directly activated by the inflammatory TFs such as NF-κb, Stat4/6, Atf-4 and Hif1; the induced JMJD3, in turn, functions as a major co-activator of these TFs in driving the transcriptional activation of numerous inflammatory genes, thus forming a positive feedback circuit facilitating the rapid induction of immunological response^3^. In a similar manner, we found that C/EBPβ directly activated JMJD3 expression, which in turn couples to the regulatory effect of JMJD3 on numerous target genes such as *JUNB*, *PU*.*1*, *P53*-*P21* axis and innate immunity components. Moreover, as mentioned above, the NF-κb-centered activation of innate immunity pathways play a determining oncogenic role in MDS and T-ALL^15,21^. Nevertheless, this study also indicates that a JMJD3-mediated activation of innate immunity, as marked by the induction of RIPK3-IL-1β axis in a NF-κB-independent manner within AML cells, is unfavorable for the maintenance of malignancy^34^.

## Methods

### Mice

NOD/SCID mice were purchased from Shanghai SLAC Laboratory Animal Co., Ltd., and were fed and maintained in accordance with the institutional guidelines for animals of Shanghai Jiao Tong University School of Medicine. All animal experiments were carried out in accordance with guidelines approved and provided by the Laboratory Animal Resource Center of Shanghai Jiao Tong University School of Medicine.

### Primary AML blasts samples, cell lines, and cell culture

Human AML samples were obtained after receiving informed consents from the patients admitted to the Department of Hematology in Ruijin Hospital. BM leukemic blasts were isolated by Ficoll-Hypaque density gradient centrifugation and cultured with Isocove modified Dulbecco medium (IMDM) supplemented with 10% BIT (Stem Cell Technologies, Vancouver, Canada) for <5 days. Leukemic cell lines (HL-60, Kasumi-1, NB4, NB4-R2, THP-1, U937, K562, and Jurkat) were obtained from Cell Bank of Shanghai Institute of Hematology and cultured in RPMI-1640 medium (Invitrogen Corporation) supplemented with 10% FBS (Gibco-BRL) in 5% CO_2_ and humidified atmosphere at 37 °C. ATRA was purchased from Sigma-Aldrich.

### Western blotting

Total cell lysates or protein extracts were equally loaded on 8–12% SDS-polyacrylamide gel for running and then transferred to polyvinylidene difluoride (PVDF) membranes (GE Healthcare-Amersham Biosciences, UK). After blocking with 5% non-fat milk in Tris buffered saline with Tween 20 (TBST), the membranes were incubated for 2 h or overnight with the primary antibodies. After staining with horseradish peroxidase (HRP)-linked secondary antibodies, signal detection was performed using a chemiluminescence phototope-HRP Kit (Millipore). The band intensity was estimated using the Photoshop Software. Densitometric analysis was performed using ImageJ software (NIH, Bethesda, MD). The following antibodies were used: anti-JMJD3 (ab154126, Abcam, 1:250), anti-C/EBPβ (sc-150×, Santa Cruz, 1:1000), anti-C/EBPα (2843, Cell Signaling, 1:1000), anti-RIPK3 (ab56164, Abcam, 1:1000), anti-JUNB (3753S, Cell Signaling, 1:1000), anti-Flag (F7425, Sigma, 1:5000) and anti-Myc (9B11, Cell Signaling, 1:1000), and anti-β-actin (A5316, Sigma, 1:5000).

### qRT-PCR and RNA-seq

Total cellular RNA was extracted using the RNeasy mini kit (QIAGEN) following the manufacturer’s instruction. The cDNA was synthesized through reverse transcription of 1 μg of RNA using RevertAid First Strand cDNA Synthesis Kit (Thermo). Quantitative real-time PCR (qRT-PCR) was performed using a 7500 sequence detection system (Applied Biosystems) and SYBR Green Realtime PCR Master Mix (Toyobo). *GAPDH* was used as an internal control in qRT-PCR. Primer sequences for qRT-PCR are available in the Supplementary Data 5.

RNA samples were sequenced and analyzed by Shanghai Majorbio Biotechnology Co., Ltd. and Shanghai Jiayin Biotechnology Co., Ltd. RNA-seq transcriptome library was prepared following TruSeqTM RNA sample preparation Kit from Illumina (San Diego, CA) using 1 μg of total RNA. Shortly, messenger RNA was isolated according to polyA selection method by oligo (dT) beads and then fragmented by fragmentation buffer firstly. Secondly, double-stranded cDNA was synthesized using a SuperScript double-stranded cDNA synthesis kit (Invitrogen, CA) with random hexamer primers (Illumina). Then the synthesized cDNA was subjected to end-repair, phosphorylation, and ‘A’ base addition according to Illumina’s library construction protocol. Libraries were size selected for cDNA target fragments of 200–300 bp on 2% Low Range Ultra Agarose followed by PCR amplified using Phusion DNA polymerase (NEB) for 15 PCR cycles. After quantified by TBS380, paired-end RNA-seq library was sequenced with the Illumina HiSeq 4000 (2 × 150 bp read length). For bioinformatics analyses, raw sequence reads were initially processed using FastQC (Babraham Institute, Cambridge, UK) for quality control, and then adapter sequences and poor quality reads were removed using Cutadapt. Quality-filtered reads were then mapped to human genome (hg19) using STAR, and only the uniquely mapped reads were kept. Read counts were calculated using HTSeq-count. Differentially expressed genes were identified using R package DESeq2 (fold change ≥2, *p* *<* 0.05 or fold change ≥1.5, *p* *<* 0.05).

### ChIP and ChIP-seq

ChIP assays were performed according to the standard cross-linking ChIP protocol (Abcam) with minor modifications. Briefly, cells were harvested and crosslinked with 1% formaldehyde for 10 min at room temperature. After sonication, the soluble chromatins were incubated with specific antibodies. Chromatin immunocomplexes were then precipitated with Protein A (Millipore, 16-661) or Protein G (Millipore, 16-662). The immunoprecipitated complex was washed, and DNA was extracted and purified by QIAquick PCR Purification Kit (Qiagen). ChIP DNA was analyzed by qPCR using specific primers, and the data were normalized by input DNA. The results were derived from three independent experiments. The primers used for ChIP-qPCR are listed in Supplementary Data 5. For ChIP-seq, extracted DNA was ligated to specific adaptors followed by deep sequencing in the Illumina HiSeq 2500 system as according to the manufacturer’s instructions. ChIP-seq data were aligned to the human genome (hg19) reference genome using bwa version 0.7.10^59^ with default parameter settings. Subsequently, reads were filtered for duplicates and extended by 200 bp. Visualization of read count data was performed by converting raw bam files to bigwig files using IGV tools^60^ and normalized to 1 million reads. For the analysis of the ChIP-seq datasets, we utilized MACS2 peak caller version 2.1.1 to identify peaks. For peak calling, we used MACS (version 2.1.1) with the sonicated input as a control and an initial threshold *q*-value of 0.01 as cutoff. For C/EBPβ ChIP-seq, signal for each gene was quantified as total number of reads per million in the region 3 kb upstream of the TSS to 3 kb downstream of the TSS. The bioinformatics analysis of ChIP-seq data was performed by Shanghai Jiayin Biotechnology Co., Ltd.

### co-IP assay

The cells were lysed in 1 ml lysis-buffer (50 mM Tris pH 7.5, 150 mM NaCl, 10% glycerol, 0.5% Triton X-100, 2 mM EDTA, 25 mM glycerophosphate, 100 mM NaF, 200 mM Na_3_VO_4_, 1 mg/ml leupeptin, 1 mg/ml aprotinin, and 1 mM PMSF). According to the manufacturer’s protocol, the insoluble material in cell lysates was removed via centrifugation, and the recovered supernatant was immunoprecipitated with Protein G-coated magnetic Dynabeads (Invitrogen) and 10 µg of each of the following antibodies separately: anti-JMJD3 (ab85392, Abcam); anti-C/EBPβ (sc-150×, Santa Cruz); anti-FLAG (F7425, Sigma), and control IgG (ab172730, Abcam). To be prepared for western blotting analysis, firstly the immunoprecipitates were washed four times with 1 ml lysis-buffer, then resuspended in 50 µl 2× Laemmli sample buffer (125 mM Tris pH 6.8, 4% SDS, 25% glycerol, 0.1% bromophenol blue, 5% β-mercaptoethanol), and finally boiled at 95 °C for 10 min. Control (INPUT) was made by 50 µl cell lysates processed for all steps except for the incubation with Protein G-coated magnetic Dynabeads and antibodies. The uncropped scans of blots are shown in the Supplementary Fig. 7.

### Flow cytometry analysis and cell sorting

All antibodies were purchased from BD PharMingen or eBiosciences (CA, USA) as follows: PE-conjugated CD11b, APC-conjugated Annexin V, and APC-conjugated Ki67. Total cells were Fc-blocked and stained with indicated combinations of antibodies for 30 min on ice, then washed three times and resuspended in 1% FBS/PBS. For apoptosis analysis, cells were resuspended with binding buffer and stained with Annexin V and 7-AAD for 15 min at 25 °C in the dark. For cell cycle analysis, cells were thoroughly suspended and incubated with fixation and permeabilization solution for 20 min at room temperature, washed twice with BD Perm/Wash buffer, and stained with KI-67 and HO33342 for 30 min. The flow cytometric data were collected on a BD Calibur or a LSRII flow cytometer and analyzed using FlowJo software or Summit software. For the fluorescence-activated cell sorting (FACS), the nucleated cells were stained with the indicated antibodies and resuspended in 2% FBS/PBS. The cells were sorted using a MoFlo machine (Beckman Coulter).

### Establishment of *JMJD3* knockout cell line

For establishing *JMJD3* knockout HL-60 cell lines, the gRNAs were designed and cloned in a U6 targeting vector^61^, and the single clones were established by dilution cloning. The knockout efficiencies were confirmed by western blotting assay. Guide RNA sequence used was: *JMJD3* exon1 5′-GGCAGCCATGCGCTACGAGG-3′.

### Lentivirus transduction and siRNA transfection

All the plasmid for packaging lentivirus, including pMD2.G and psPAX2, were purchased from Addgene (Cambridge, MA). Firstly, 5 μg pMD2.G, 10 μg psPAX2, and 6 μg empty vector plasmids (pLVX-IRES-GFP or pLVX-IRES-YFP) or 12 μg JMJD3 constructs (pLVX-IRES-GFP-JMJD3-WT or pLVX-IRES-YFP-JMJD3-WT) or catalytically inactive JMJD3 mutant (pLVX-IRES-GFP-JMJD3-H1390A) were co-transfected into HEK-293T cells in 100 mm cell culture dish with Sofast Effectene Transfection Reagent (Sunmabio, China). The lentiviral particles were harvested at 48 and 72 h after transfection and concentrated with XE-90 Super Speed Centrifuger (BECKMAN). The lentiviruses were subsequently used to infect leukemic cell lines or primary AML blast samples^62^. Leukemia cells or primary AML blasts were counted and spin-infected with lentiviral particles. Finally, leukemia cells were washed with PBS 12 h after infection. GFP^+^ cells were isolated by FACS after 3 days and sorted again after 5 days; primary AML blasts were washed with PBS 12 h after infection. GFP^+^ cells were isolated by FACS after 2 days. For C/EBPβ overexpression, HL-60 cells were spin-infected with lentivirus containing empty control vector (pLVX-IRES-tdTomato) or C/EBPβ (pLVX-IRES-tdTomato-C/EBPβ), tdTomato^+^ cells were isolated by FACS after 3 days and sorted again after 1 week.

*C/EBPβ*, *RIPK3*, and *JUNB* siRNAs (Genomeditech, China) were transfected into HL-60 cells at a concentration of 50 nM using an electroporation system (GP7901, Genloci, China) according to the manufacturer’s instructions. The following siRNA sequences were used in this study: *C/EBPβ*-siRNA-1, sense, 5′-GCCCUGAGUAAUCACUUAAAGTT-3′; *C/EBPβ*-siRNA-1, antisense, 5′-CUUUAAGUGAUUACUCAGGGCTT-3′; *C/EBPβ*-siRNA-2, sense, 5′-GUAUAUUUUGGGAAUCUUUTT-3′; *C/EBPβ*-siRNA-2, antisense, 5′-AAAGAUUCCCAAAAUAUACTT-3′; *C/EBPβ*-siRNA-3, sense, 5′-GGCCCUGAGUAAUCGCUUATT-3′; *C/EBPβ*-siRNA-3, antisense, 5′-UAAGCGAUUACUCAGGGCCTT-3′; *RIPK3*-siRNA-1, sense, 5′-CCAGCACUCUCGUAAUGAUTT-3′; *RIPK3*-siRNA-1, antisense, 5′-AUCAUUACGAGAGUGCUGGTT-3′; *RIPK3*-siRNA-2, sense, 5′-CCGGCUUAGAAGGACUGAATT-3′; *RIPK3*-siRNA-2, antisense, 5′-UUCAGUCCUUCUAAGCCGGTT-3′; *RIPK3*-siRNA-3, sense, 5′-GAACUGUUUGUUAACGUAATT-3′; *RIPK3*-siRNA-3, antisense, 5′-UAUAUGUUAACGAGCGGUCTT-3′; *JUNB*-siRNA-1, sense, 5′-CUCUCUACACGACUACAAATT-3′; *JUNB*-siRNA-1, antisense, 5′-UUUGUAGUCGUGUAGAGAGTT-3′; *JUNB*-siRNA-2, sense, 5′-CGCCGACGGCUUUGUCAAATT-3′; *JUNB*-siRNA-2, antisense, 5′-UUUGACAAAGCCGUCGGCGTT-3′; *JUNB*-siRNA-3, sense, 5′-AGACCAAGAGCGCAUCAAATT-3′; *JUNB*-siRNA-3, antisense, 5′-UUUGAUGCGCUCUUGGUCUTT-3′. *JMJD3*-siRNA-1, sense, 5′-CGCUGACCAUUACCAAACUTT-3′; *JMJD3*-siRNA-1, antisense, 5′-AGUUUGGUAAUGGUCAGCGTT-3′; *JMJD3*-siRNA-2, sense, 5′-GUGACAAGGAGACCUUUAUTT-3′; *JMJD3*-siRNA-2, antisense, 5′-AUAAAGGUCUCCUUGUCACTT-3′; *JMJD3*-siRNA-3, sense, 5′-GAGACCUCGUGUGGAUUAATT-3′; *JMJD3*-siRNA-3, antisense, 5′-UUAAUCCACACGAGGUCUCTT-3′; *JMJD3*-siRNA-4, sense, 5′-GCGAUGUGGAGGUGUUUAATT-3′; *JMJD3*-siRNA-4, antisense, 5′-UUAAACACCUCCACAUCGCTT-3′.

### Human AML repopulation assay

HL-60 cells were transplanted into NOD-SCID mice by intravenously injection^63^. Briefly, HL-60 cells were transduced with control, JMJD3-, or H1390A mutant-expressing lentiviral vector and sorted out by FACS. Ten-week-old female NOD-SCID mice were sublethally irradiated with 300 cGy and then intravenously injected with 5 × 10^6^ transduced HL-60 cells resuspended in 200 µl PBS containing 1% FBS. About 35 days after transplantation, the PB, BM, spleen, and liver were assessed for the leukemic repopulation.

### Leukemia cell transplantation

GFP^+^ BM leukemic blasts expressing the hPML-RARα-, hAML1-ETO9a-, or hMLL-AF9 were first infected with pLVX-IRES-YFP or pLVX-IRES-YFP-JMJD3-WT lentiviral vector during a short-term in vitro culture, then the GFP^+^YFP^+^ leukemic cells were transplanted into syngeneic mice to allow them to repopulate in vivo. For leukemic cells derived from hPML-RARα-transgenic mice, 6–8-week-old female FVB/NJ mice were sublethally irradiated with 400 cGy and then intravenously injected with 1 × 10^6^ transduced leukemic cells resuspended in 200 μl PBS. For leukemic cells expressing hAML1-ETO9a or hMLL-AF9, 6–8-week-old female C57BL/6J mice were sublethally irradiated with 400 cGy and then intravenously injected with 1 × 10^6^ transduced leukemic cells resuspended in 200 µl PBS.

### **In vitro** colony-formation assays

Primary AML blasts samples transduced with control or JMJD3-overexpressed vector were diluted and seeded at 50,000 per 35-mm dishes in methylcellulose medium (MethoCult™ H4435, Stem Cell Technologies). HL-60 cells transduced with control or JMJD3-overexpressed vector were further transfected with NC or *C/EBPβ* siRNA, these cells were diluted and seeded at 5000 per 35-mm dishes in methylcellulose medium (MethoCult™ M3434, Stem Cell Technologies). The number of colonies with more than 50 cells was counted after 2 weeks.

### Bioinformatics analyses with GEO and TCGA data

The microarray expression data in BM and PB mononuclear cells of AML patients (including seven BM or 19 PB samples) and in normal counterparts (ten BM and ten PB samples) were obtained from the GEO GSE9476 dataset^64^. Data were first normalized using Robust Multi-array Average (RMA) across samples^65^. The unpaired Student’s *t* test per gene probe was used to determine the significant difference between sample categories. The mRNA expression data and clinical information of AML samples were obtained from a patient cohort reported by Verhaak and colleagues^22^. For AML patients, we correlated the expression levels with the outcome of patients, and observed that boundaries defined as 0–45% (low) and 55–100% (high) of *JMJD3* expression predicted outcome of patients. For AML patients with M0, M1, M2, and M3 subtypes, or AML patients with M4 and M5 subtypes, boundaries defined as 0–50% (low) and 50–100% (high) of *JMJD3* expression predicted outcome of patients. Survival analysis was performed using Kaplan–Meier analysis and the log-rank test was used to assess the statistical significance. The mRNA expression data of 179 AML samples were obtained from TCGA AML database^66^, for correlation analysis, scatter plots with regression curves (blue), and 95% prediction intervals (red) indicate the positive correlations among *JMJD3, C/EBPβ*, or *RIPK3*. Correlation coefficient *r* and *p* values are shown.

### Gene-set enrichment analysis and gene ontology analysis

GSEA was performed using the Broad Institute web platform by pre-ranking the RNA-seq list based on log2-fold change. Gene ontology analysis was performed with DAVID tools (http://david.abcc.ncifcrf.gov/tools.jsp).

### H3K4me3/H3K27me3 gain and loss analyses

Histone modification status changes between the *JMJD3* knockout and parental HL-60 cells were determined across the genome using the following method. Briefly described, the total numbers of reads of H3K4me3/H327me3 around gene TSSs (±3 kb) were calculated. For each gene, the fold change between the two samples was calculated (*JMJD3* knockout versus parental) and greater than 1.5 is defined as significant epigenetic change.

### ChIP-seq data analysis

AML1-ETO ChIP-seq data for Kasumi-1 cells were obtained from ref. ^26^ (GEO accession: GSE65427), PML and RARα ChIP-seq data for NB4 and UPR9 cells were obtained from ref. ^27^ (GEO accession: GSE18886). MLL-AF9 ChIP-seq data for leukemic blast cells were obtained from the published work by Bernt et al. (GEO accession: GSE29130)^28^. ChIP-seq data were processed by Cistrome analysis pipeline and were loaded in UCSC genome browsers for visualization^67^.

### Statistical analysis

Microsoft Excel and GraphPad Prism softwares were used for statistical analysis. Two-tailed Student’s *t* test was used for statistical analysis unless otherwise indicated. The Kaplan–Meier method was used to plot survival curves and the log rank test was used to evaluate statistical differences. The hypergeometric test was performed in R to calculate the statistical significance of overlapped genes illustrated by Venn diagrams. A *p* value of less than 0.05 was considered statistically significant. Data are presented as mean ± SEM.

### Data availability

The accession number for the raw data of RNA-seq and ChIP-seq reported in this paper is GEO: GSE101891. All other relevant data are available from the corresponding author on request.

## Electronic supplementary material

Supplementary Information

Description of Additional Supplementary Files

Supplementary Data 1

Supplementary Data 2

Supplementary Data 3

Supplementary Data 4

Supplementary Data 5
