# Supplementary material for: JMJD3 facilitates C/EBPβ-centered transcriptional program to exert oncorepressor activity in AML
Source: Nat Commun. 2018 Aug 22;9:3369. doi: 10.1038/s41467-018-05548-z (PMC6105679; doi:10.1038/s41467-018-05548-z)
Supplement: Supplementary file 1 — Supplementary Information [file 41467_2018_5548_MOESM1_ESM.pdf]

Supplementary Information

JMJD3 facilitates C/EBPβ-centered transcriptional program to exert oncorepressor activity in AML

Yu et al.

Suplementary Figure 1

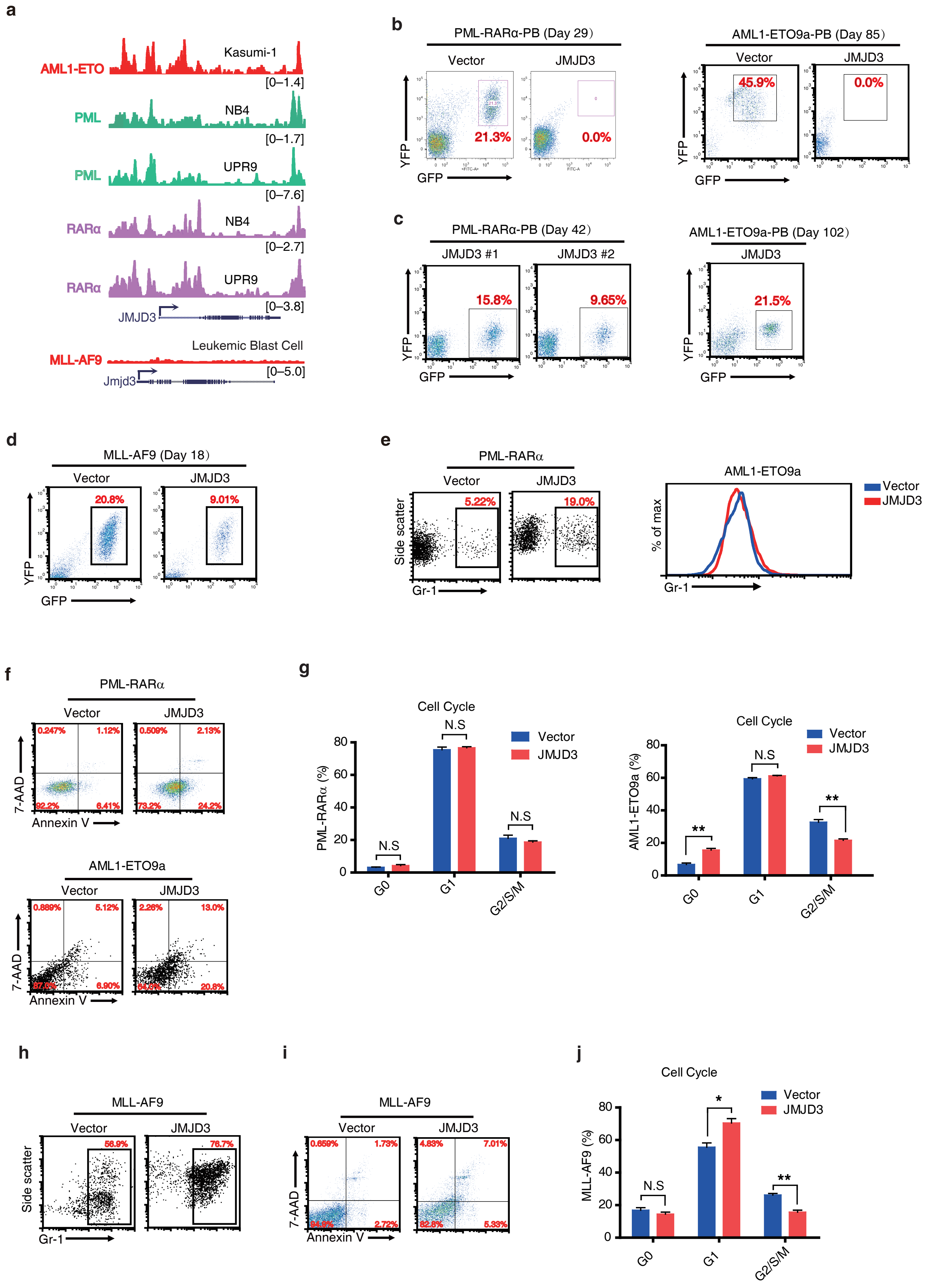

Supplementary Figure 1. Characterization of JMJD3 oncorepressor activity in three mouse AML models, related to Figure 2.

(a) Genome browser tracks representing the binding sites of AML1-ETO, PML, RARα or MLL-AF9 at the *JMJD3* gene locus in the AML cells as indicated. (b) The representative flow cytometric analyses on the percentages of PML-RARα$^+$ or AML1-ETO9a$^+$ GFP$^+$YFP$^+$ cells in the peripheral blood of the recipients after they had been transduced with empty vector or JMJD3-expressing vector. (c) The percentages of PML-RARα$^+$ (left panel) or AML1-ETO9a$^+$ (right panel) GFP$^+$ leukemia cells in the peripheral blood of syngeneic recipients. (d) The percentages of MLL-AF9$^+$GFP$^+$YFP$^+$ cells in the peripheral blood of syngeneic mice after the leukemia cells had been transduced with empty vector or JMJD3-expressing vector. (e-g) Flow cytometric assay of Gr-1 expression (e), Annexin V/7-AAD staining (f) or cell-cycle status (g) on PML-RARα$^+$ or AML1-ETO9a$^+$ leukemia BM cells transduced with YFP$^+$ empty vector or JMJD3-expressing vector. (h-j) Flow cytometric analyses of CD11b expression (h), Annexin V/7-AAD staining (i) and cell cycles (j) on MLL-AF9$^+$GFP$^+$YFP$^+$ cells in the BM of syngeneic recipients after the leukemia cells had been transduced with empty vector or JMJD3-expressing vector. Data are shown as the mean ± SEM. *$p < 0.05$, **$p < 0.01$.

Suplementary Figure 2

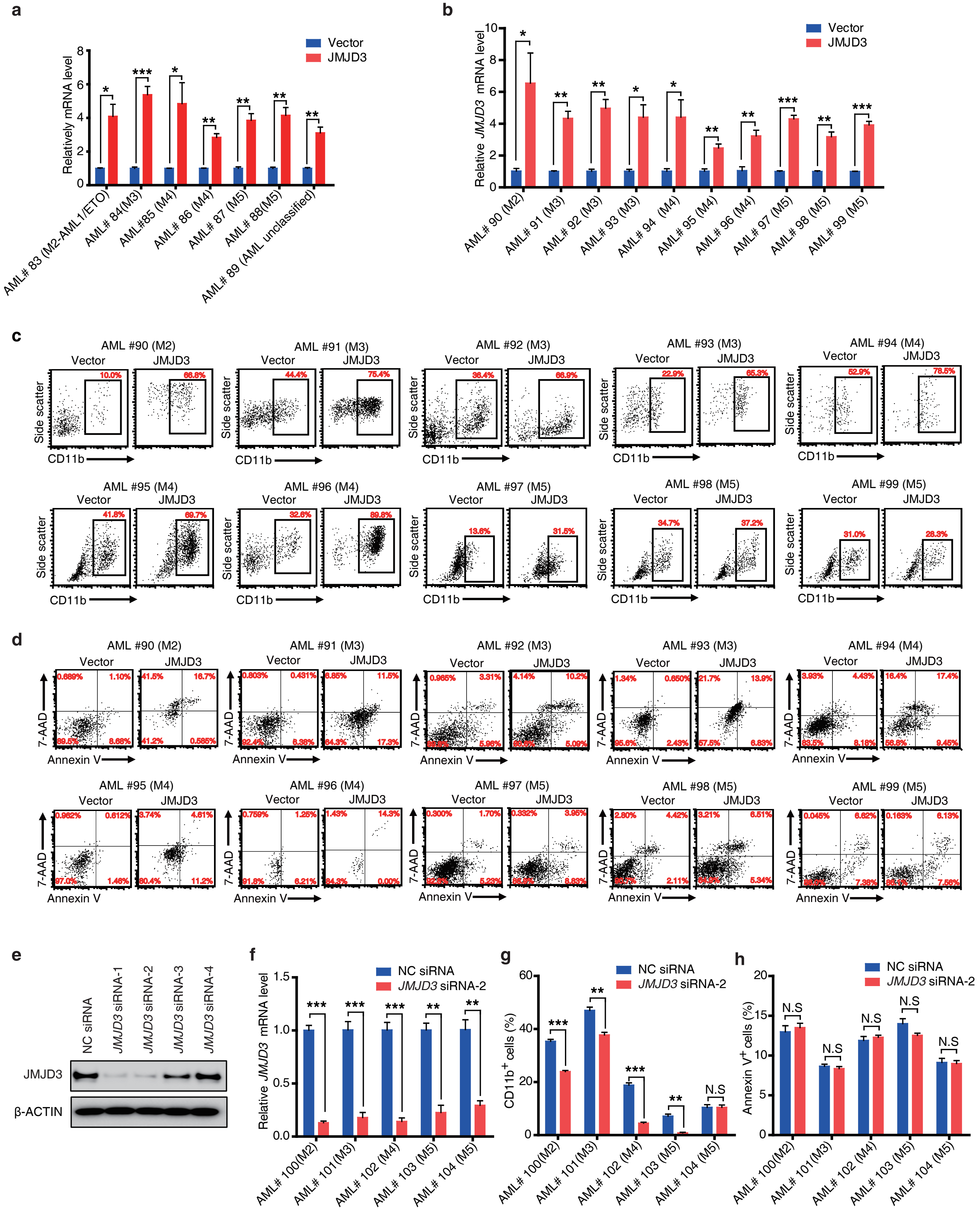

Supplementary Figure 2. JMJD3 exhibits oncorepressor activity in the primary leukemic blasts, related to Figure 3.

(a) qRT-PCR expression analysis of *JMJD3* mRNA level on 7 primary AML blasts transduced with control or JMJD3-expressing vector. (b) qRT-PCR expression analysis of *JMJD3* mRNA level on 10 primary AML blasts transduced with control or JMJD3-expressing vector. (c-d) Analyses of CD11b expression (c) or Annexin V/7-AAD staining (d) on 10 primary AML blasts transduced with control or JMJD3-expressing vector using flow cytometric assay. (e) Western blotting assay on JMJD3 protein level in 293T cells transduced with NC siRNA or *JMJD3* siRNA. (f) qRT-PCR expression analysis of *JMJD3* mRNA level on 5 primary AML blasts transduced with NC siRNA or *JMJD3* siRNA. (g-h) Analyses of CD11b expression (g) or Annexin V staining (h) on 5 primary AML blasts transduced with NC siRNA or *JMJD3* siRNA. Data are shown as the mean ± SEM. *$p < 0.05$, **$p < 0.01$, ***$p < 0.001$.

Suplementary Figure 3

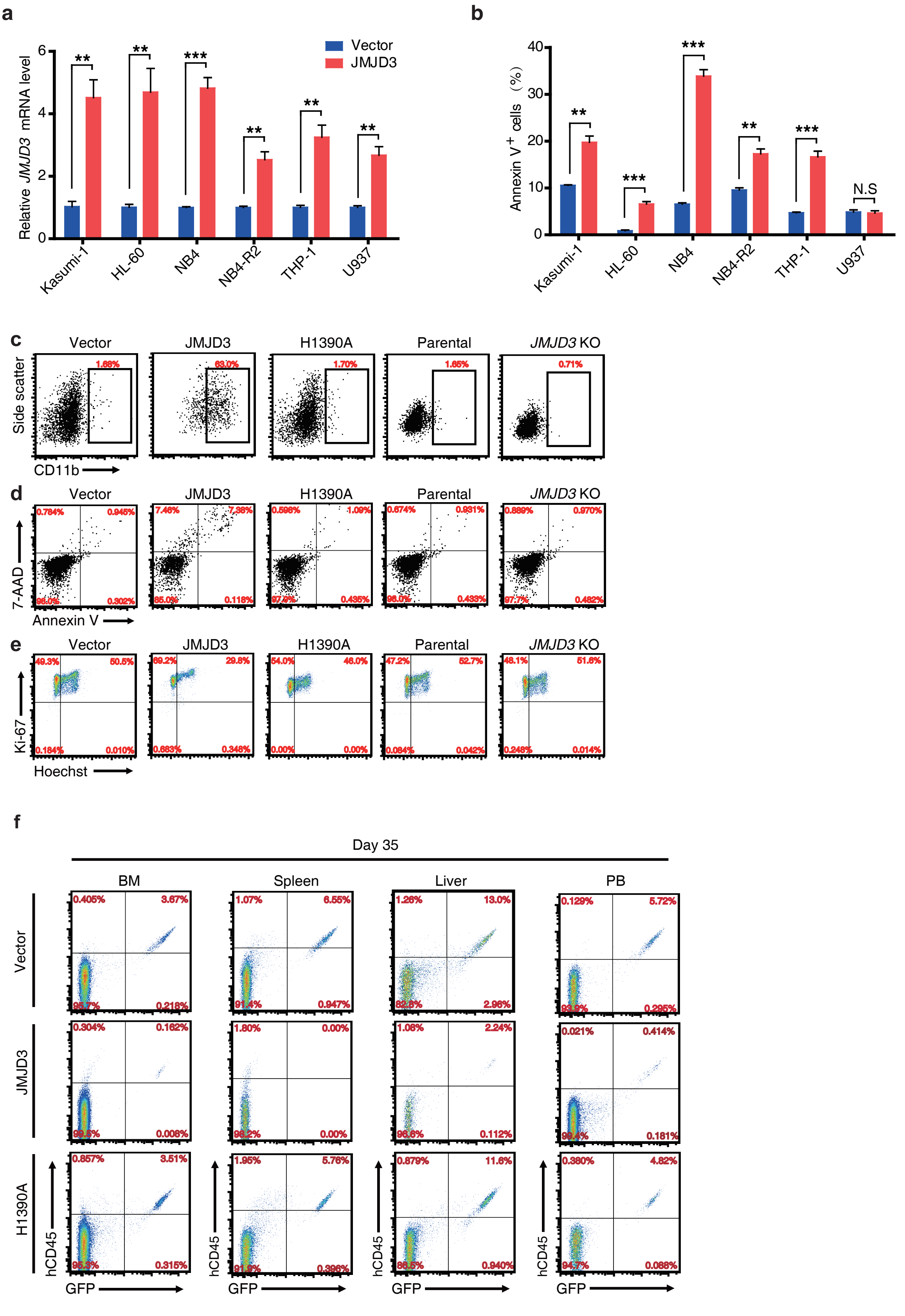

Supplementary Figure 3. JMJD3 exhibits oncorepressor activity in the established human AML cell lines, related to Figure 3.

(a) qRT-PCR analyses of *JMJD3* mRNA level on the established AML cells transduced with control or JMJD3-expressing vector. (b) Flow cytometric analyses of Annexin V staining on the established AML cells transduced with control or JMJD3-expressing vector. (c-e) Flow cytometric assay of CD11b expression (c), Annexin V/7-AAD staining (d) or Hoechst/KI-67 staining (e) on HL-60 cells transduced with vector control, JMJD3- or H1390A mutant-expressing vector, or in parental or *JMJD3* knockout HL-60 cells. (f) Flow cytometry plots indicating BM, Spleen, Liver and PB engraftment of HL-60 cells transduced with vector control, JMJD3- or H1390A mutant-expressing vector on the day 35 after inoculation. The co-staining with anti-human CD45 confirmed that the engraftment was from transplanted human leukemia cells. Data are shown as the mean ± SEM. $^{**}p < 0.01$, $^{***}p < 0.001$.

Suplementary Figure 4

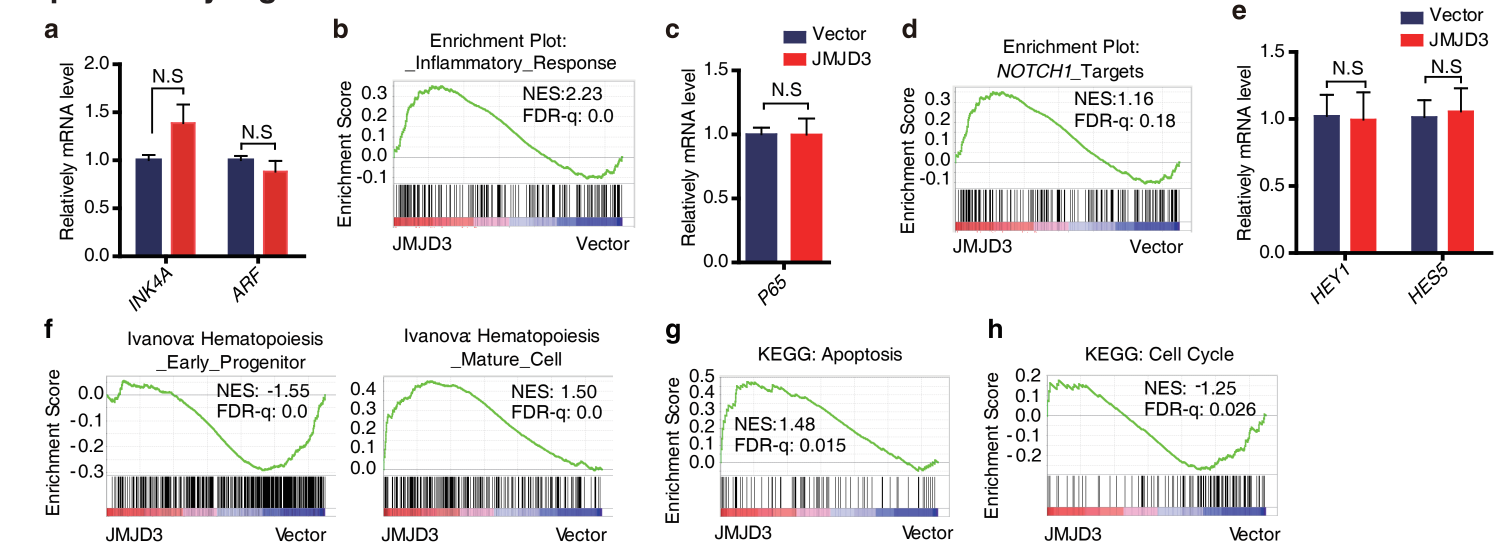

i

	GO Term	Count	%	*p* value
1	leukocyte activation	109	15.7	1.67E-14
2	leukocyte differentiation	65	14.3	1.30E-07
3	leukocyte proliferation	41	15.3	5.59E-06
4	hemopoiesis	85	11.8	8.20E-06
5	neutrophil activation	8	34.8	0.000148196
6	myeloid cell differentiation	42	12.5	0.000512154
7	myeloid cell apoptotic process	6	21.4	0.014318323
8	myeloid cell development	8	15.4	0.034500118
9	macrophage differentiation	6	16.7	0.045095131

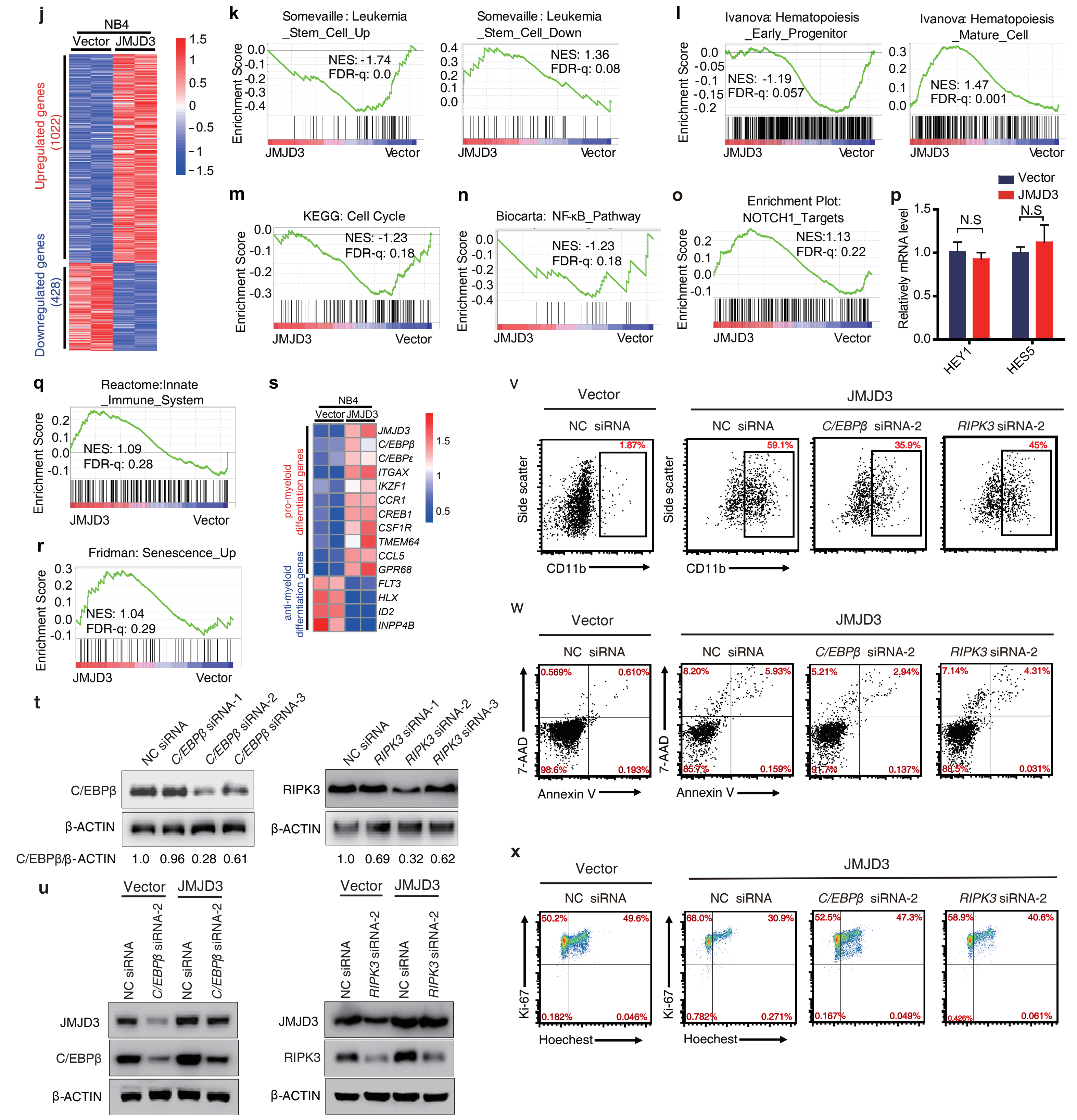

Supplementary Figure 4. JMJD3 modulated the expression of key myelopoietic genes, related to Figure 4.

(a) qRT-PCR expression analysis of *INK4A* and *ARF* mRNA level on HL-60 cells transduced with control or JMJD3-expressing vector. (b) GSEA of the expressing profile of HL-60 cells transduced with control or JMJD3-expressing vector using an inflammatory response-associated signature. (c) qRT-PCR expression analysis of the mRNA level of the *P65* subunit of NF-κB on HL-60 cells transduced with control or JMJD3-expressing vector. (d) GSEA of the expressing profile of HL-60 cells transduced with control or JMJD3-expressing vector using a NORTCH1 targets-associated signature. (e) qRT-PCR expression analysis of *HEY1* and *HES5* mRNA level on HL-60 cells transduced with control or JMJD3-expressing vector. (f-h) GSEA of the expressing profile of HL-60 cells transduced with control or JMJD3-expressing vector using a hematopoietic early progenitor-associated signature (f, left panel), a hematopoietic mature cell-associated signature (f, right panel), an apoptosis-associated signature (g) and a cell cycle-associated signature (h). (i) Summary of the top functional categories of genes significantly enriched in HL-60 cells transduced with JMJD3-expressing vector. Analyses were performed on the upregulated genes in HL-60 cells by JMJD3 overexpression using DAVID. (j) Heatmap showing the differentially expressed genes between NB4 cells transduced with control or JMJD3-expressing vector (fold change ≥2, $p < 0.05$). (k-o) GSEA of the expressing profile of NB4 cells transduced with control or JMJD3-expressing

vector using a leukemic stem cell (LSC)-associated upregulated signature (k, left panel), a LSC-associated downregulated signature (k, right panel), a hematopoietic early progenitor-associated signature (l, left panel), a hematopoietic mature cell-associated signature (l, right panel), a cell cycle-associated signature (m), a NF-κB pathway-associated signature (n) and a NORTCH1 targets-associated signature (o). (p) qRT-PCR expression analysis of *HEY1* and *HES5* mRNA level on NB4 cells transduced with control or JMJD3-expressing vector. (q-r) GSEA of the expressing profile of NB4 cells transduced with control or JMJD3-expressing vector using an innate immunity-associated signature (q) and a senescence-associated signature (r). (s) Heatmap showing the differentially expressed genes between NB4 cells transduced with control or JMJD3-expressing vector (fold change ≥1.5, $p < 0.05$) on a number of pro-myeloid differentiation genes and anti-myeloid differentiation genes. (t) Western blotting assay on C/EBPβ or RIPK3 protein in HL-60 cells transduced with NC siRNA, *C/EBPβ* siRNA or *RIPK3* siRNA. (u) Western blotting assay on JMJD3 and C/EBPβ protein for *C/EBPβ* knockdown experiments in HL-60 cells transduced with control or JMJD3-expressing vector (left panel), western blotting assay on JMJD3 and RIPK3 protein for *RIPK3* knockdown experiments in HL-60 cells transduced with control or JMJD3-expressing vector (right panel). (v-x) Flow cytometric assay of CD11b expression (v), Annexin V/7-AAD staining (w) and Hoechst/KI-67 staining (x) for *C/EBPβ* or *RIPK3* knockdown experiments in HL-60 cells transduced with control or JMJD3-expressing vector.

Suplementary Figure 5

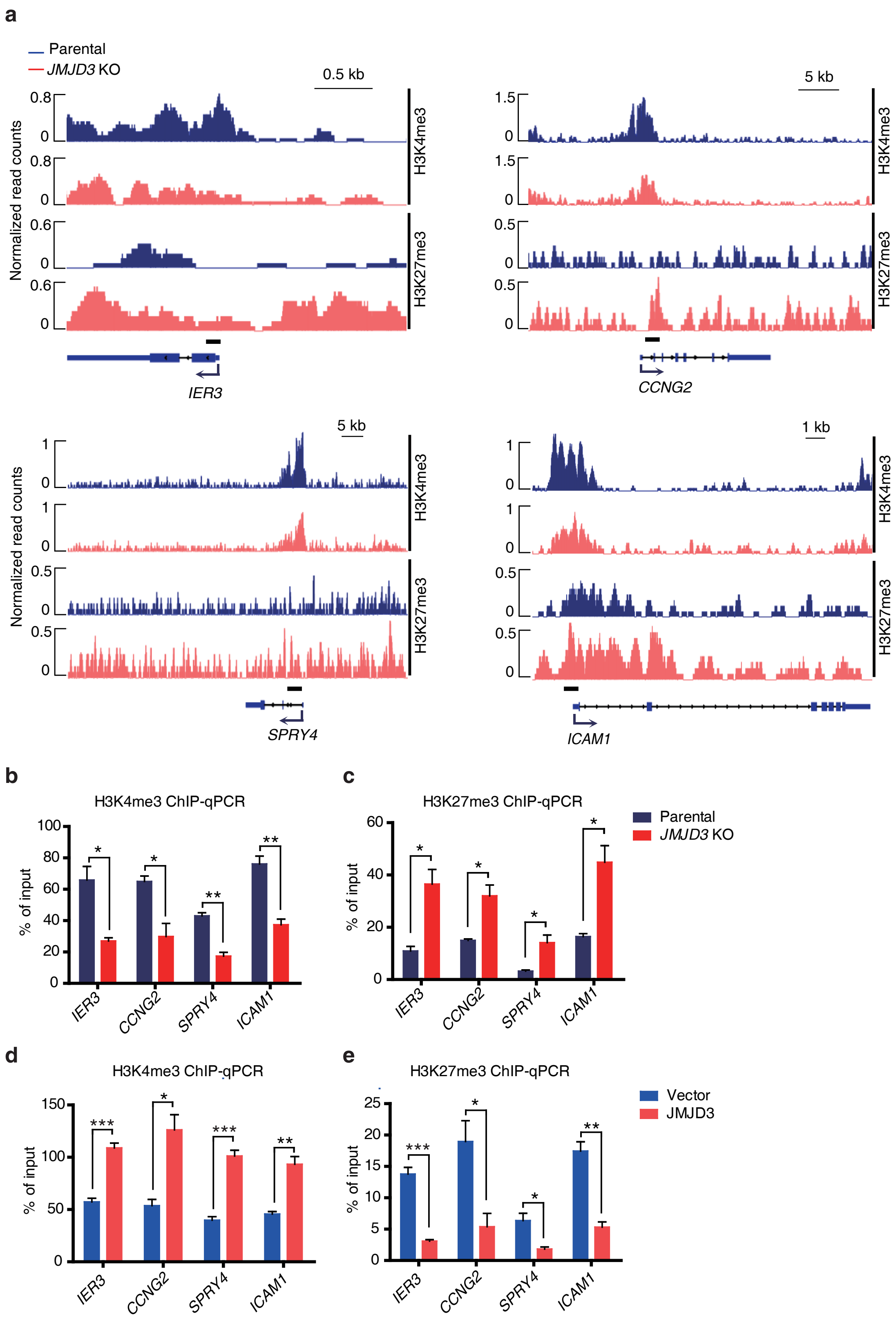

Supplementary Figure 5. JMJD3 modulated the H3K4/K27 methylation in the promoter of myelopoietic regulators, related to Figure 5.

(a) Genome browser tracks representing the binding sites of H3K4me3 and H3K27me3 at four representative gene locus in parental and *JMJD3* knockout HL-60 cells. (b-c) ChIP-qPCR assay for H3K4me3 (b) and H3K27me3 (c) at four representative gene loci in parental and *JMJD3* knockout HL-60 cells. (d-e) ChIP-qPCR assay for H3K4me3 (d) and H3K27me3 (e) at four representative gene loci in HL-60 cells transduced with control or JMJD3-expressing vector. Data are shown as the mean ± SEM. *$p < 0.05$, **$p < 0.01$, ***$p < 0.001$.

Suplementary Figure 6

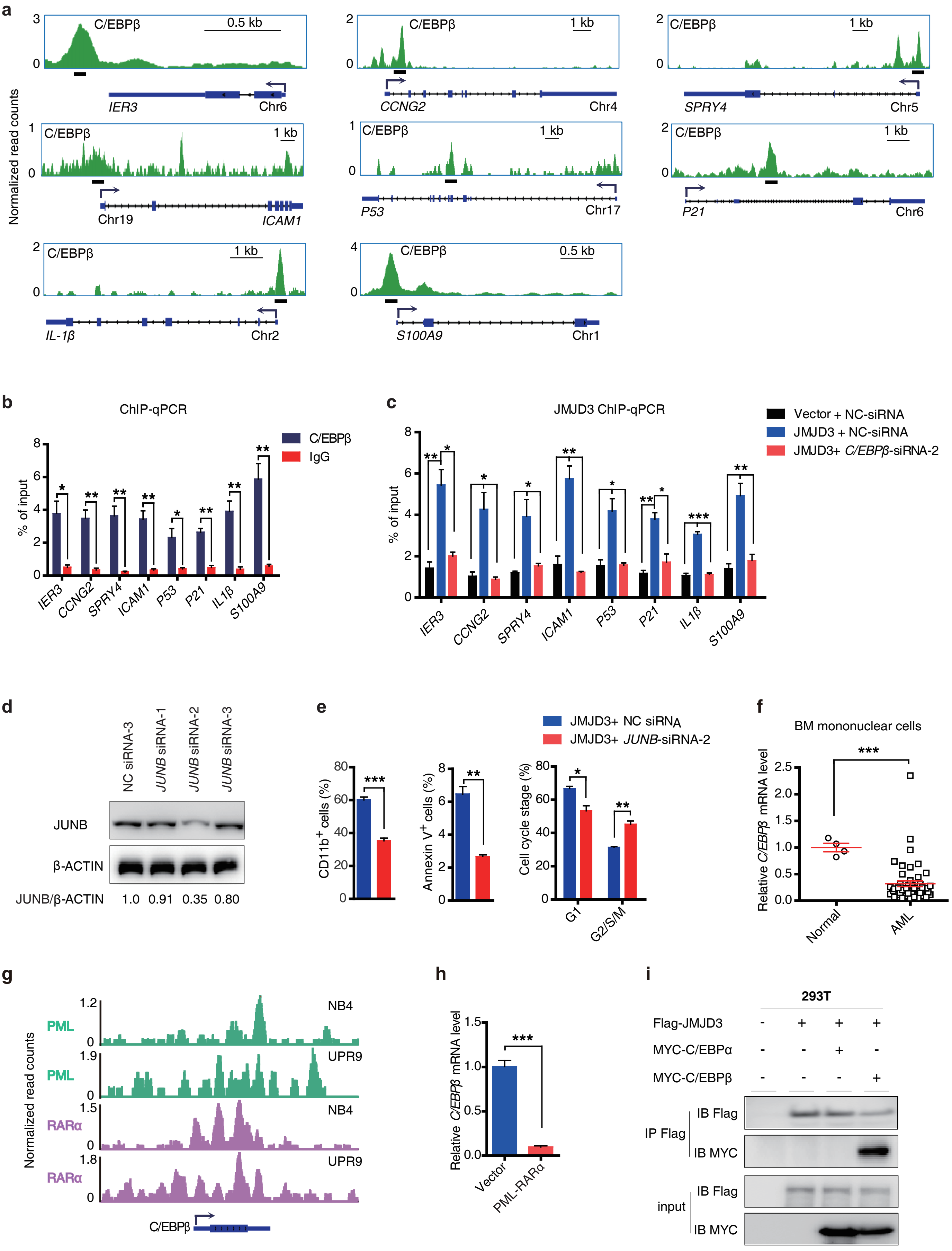

Supplementary Figure 6. JMJD3 couples with C/EBPβ program, related to Figure 6.

(a) Genome browser tracks representing the binding sites of C/EBPβ at 8 representative gene loci. (b) ChIP-qPCR assay for C/EBPβ occupancy at 8 representative gene loci in HL-60 cells transduced with C/EBPβ-expressing vector. (c) ChIP-qPCR assay for JMJD3 occupancy at the 8 representative gene loci for *C/EBPβ* or NC knockdown experiments in HL-60 cells transduced with control or JMJD3-overexpressing vector. (d) Western blotting assay on JUNB protein in HL-60 cells transduced with NC siRNA or *JUNB* siRNA. (e) HL-60 cells transduced with control or JMJD3-expressing vector were further treated with NC siRNA or *JUNB* siRNA. Analyses of CD11b expression (left panel), Annexin V staining (middle panel) and cell cycle status (right panel) were performed using flow cytometric assay. (f) *C/EBPβ* mRNA level in the AML blasts-enriched primary BM mononuclear cells (n=49) or normal BM mononuclear cells (n=4) were assayed by qRT-PCR. (g) Genome browser tracks representing the binding sites of PML or RARα at the *C/EBPβ* gene locus in the AML cells as indicated. (h) PML-RARα was transduced into normal c-Kit^{+} BM cells by retrovirus infection, and the mRNA levels of *C/EBPβ* were measured by qRT-PCR. (i) 293T cells were transfected with human Flag-JMJD3 and MYC-C/EBPα or MYC-C/EBPβ constructs. Cell lysates were immunoprecipitated with anti-Flag beads, followed by immunoblot with an anti-MYC antibody. Data are shown as the mean ± SEM. *$p < 0.05$, **$p < 0.01$, ***$p < 0.001$.

Figure 1d

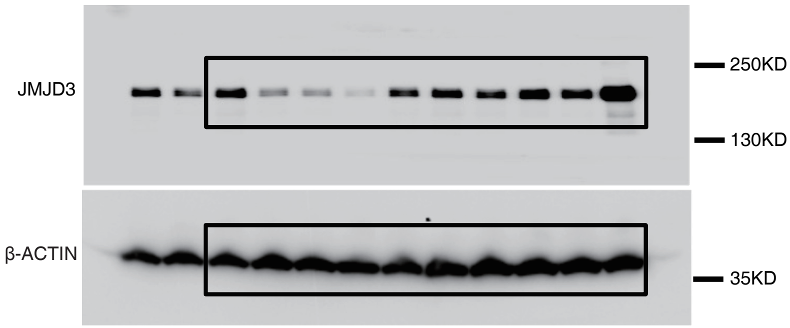

Figure 1e

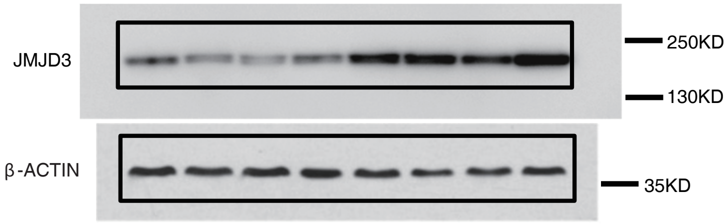

Figure 2a

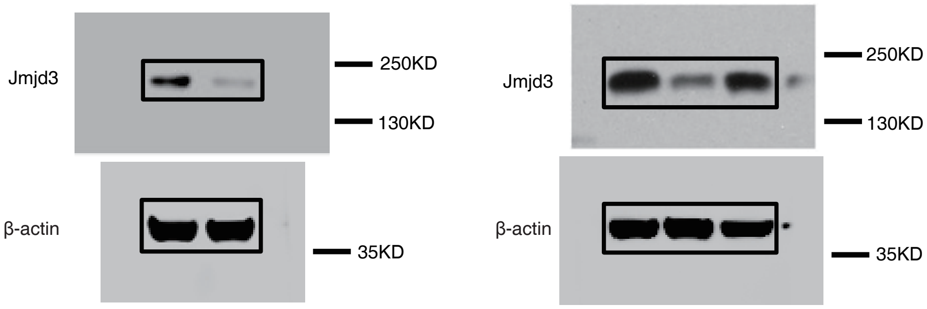

Figure 3e

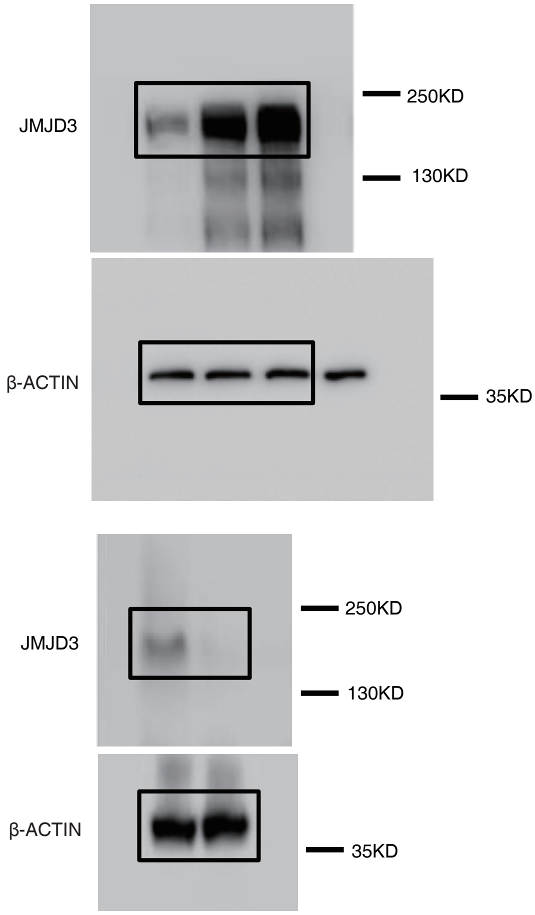

Figure 6m

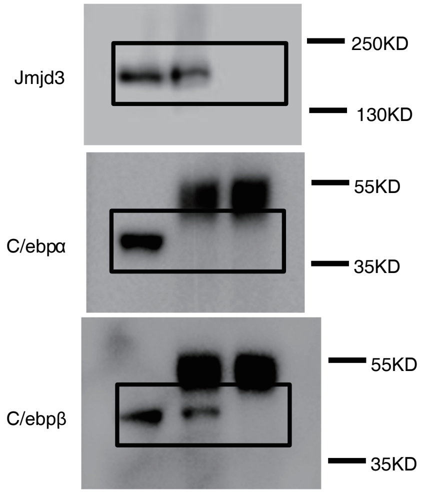

Supplementary Figure 7. The uncropped scans of blots

Figure 7a

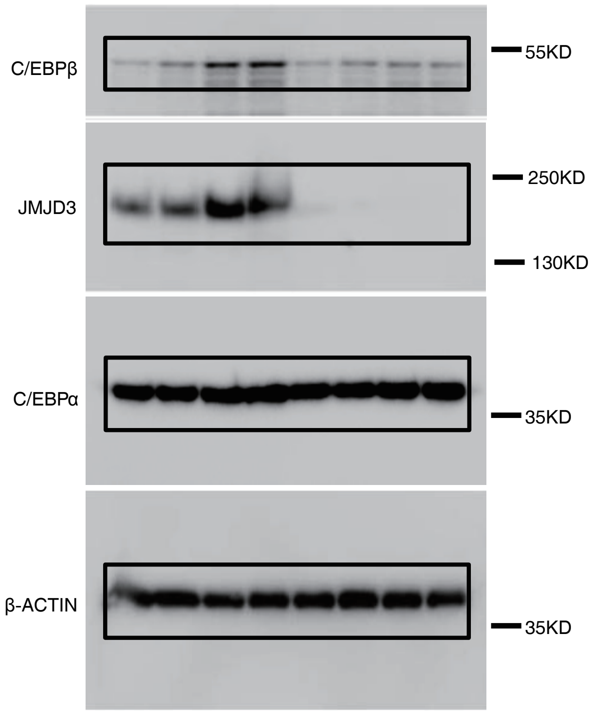

Figure 7c

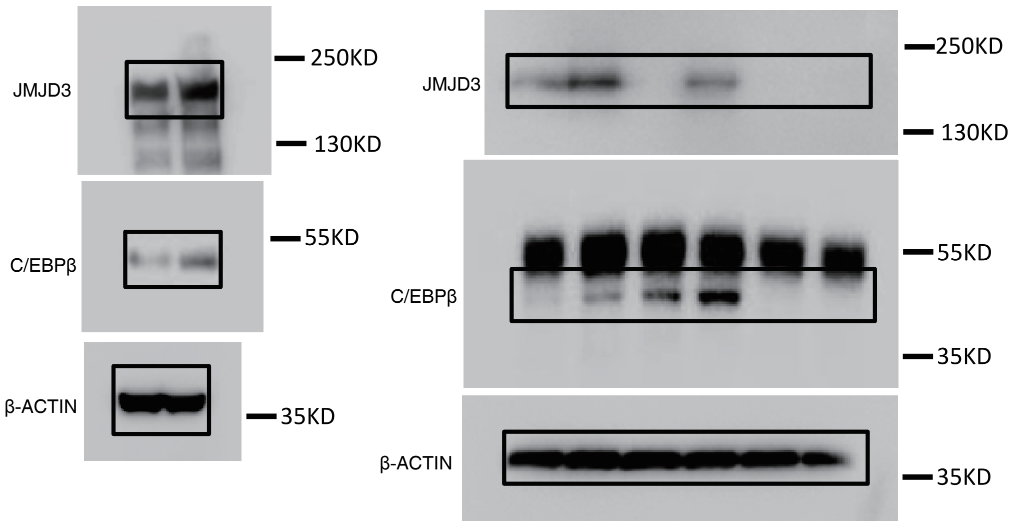

Supplementary Figure 7 continued

Supplementary Figure 2e

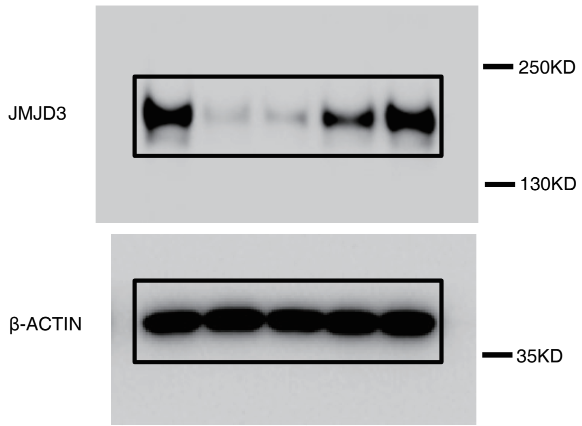

Supplementary Figure 4t

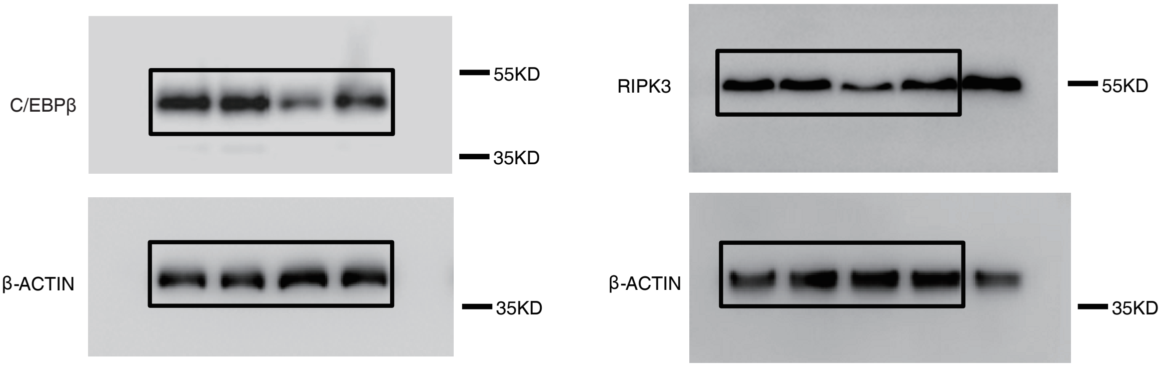

Supplementary Figure 4u

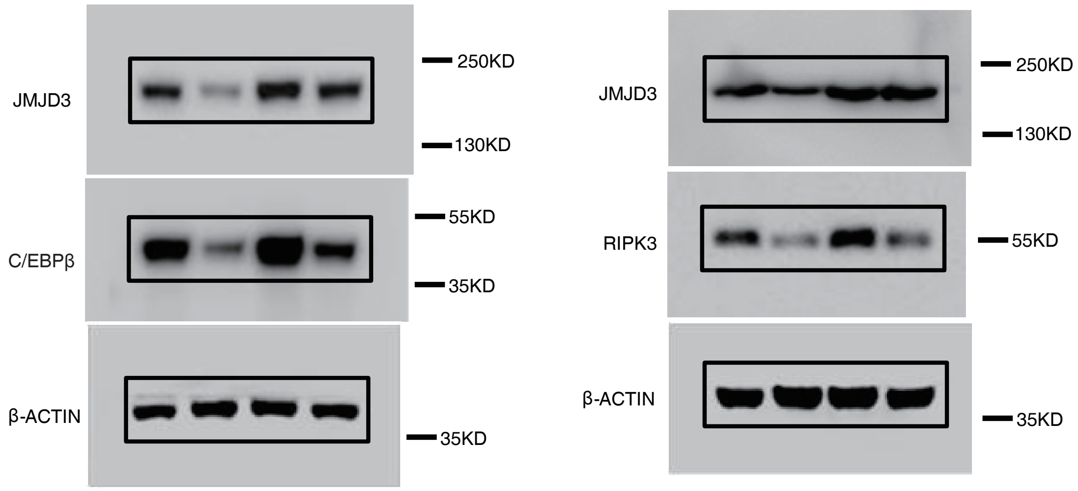

Supplementary Figure 7 continued

Supplementary Figure 6d

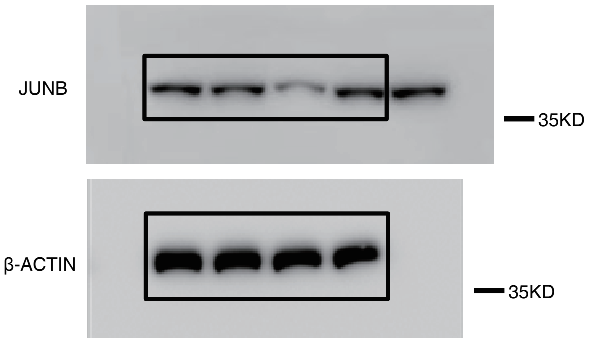

Supplementary Figure 6i

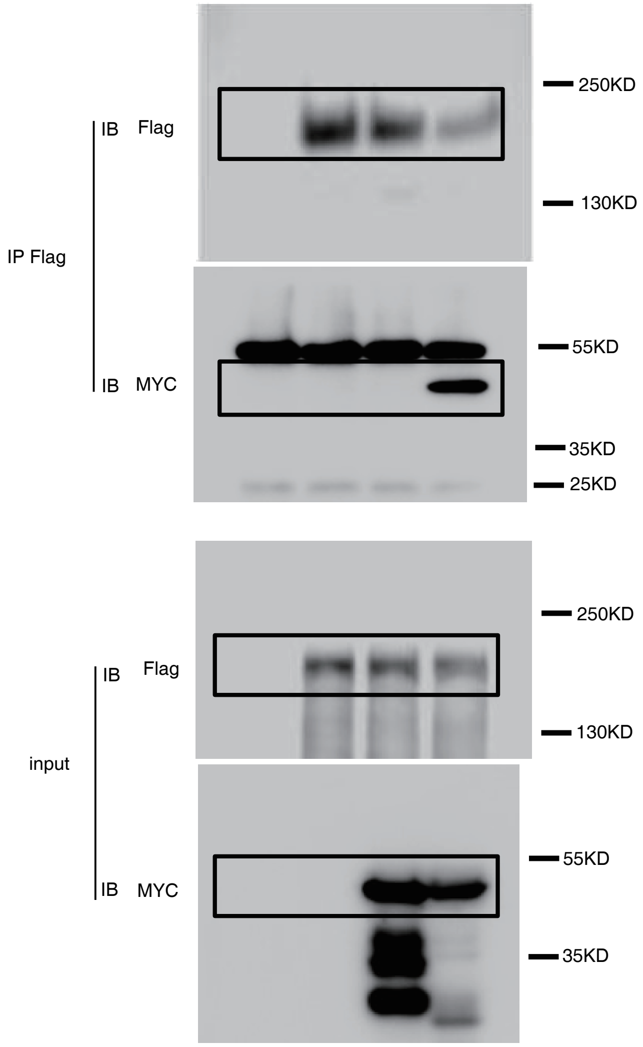

Supplementary Figure 7 continued
