# Supplementary material for: JMJD3 facilitates C/EBPβ-centered transcriptional program to exert oncorepressor activity in AML
Source: Nat Commun. 2018 Aug 22;9:3369. doi: 10.1038/s41467-018-05548-z (PMC6105679; doi:10.1038/s41467-018-05548-z)
Supplement: Supplementary file 2 — Description of Additional Supplementary Files [file 41467_2018_5548_MOESM2_ESM.pdf]

Description of Additional Supplementary Files

File Name: Supplementary Data 1
Description: Patients' information.

File Name: Supplementary Data 2
Description: Differential expression analyses of RNA-seq data between HL-60 cells transduced with control and JMJD3-expressing vector.

File Name: Supplementary Data 3
Description: Differential expression analyses of RNA-seq data between parental and *JMJD3* KO HL-60 cell lines.

File Name: Supplementary Data 4
Description: List of C/EBPβ target genes.

File Name: Supplementary Data 5
Description: Sequence of PCR primers.
